# Effectiveness of Whole‐Body Vibration Therapy on Handgrip Strength: A Systematic Review and Meta‐Analysis of Randomized Controlled Trials

**DOI:** 10.1002/msc.70246

**Published:** 2026-06-29

**Authors:** Julien Dines Labarrere, Raphael Gonçalves de Oliveira, André Luiz de Campos Pessoa, Danúbia Cunha Sá‐Caputo, Mario Bernardo‐Filho, Liszt Palmeira de Oliveira

**Affiliations:** ^1^ Universidade do Estado do Rio de Janeiro Rio de Janeiro Brazil; ^2^ Universidade Estadual do Norte do Paraná Jacarezinho Brazil

**Keywords:** exercise, mechanical vibration, muscle strength, physical fitness, rehabilitation

## Abstract

**Introduction:**

Although whole‐body vibration (WBV) therapy has already demonstrated efficacy in improving lower‐limb muscle strength, it is still unclear whether it has similar potential for improving handgrip strength.

**Objective:**

To verify the efficacy of WBV on handgrip strength.

**Methods:**

The search was conducted on September 5, 2025, in PubMed, Embase, CENTRAL, CINAHL, SPORTDiscus, Web of Science, LILACS, PEDro, and SciELO. Risk of bias was assessed via the PEDro scale, and certainty of evidence using GRADE. Meta‐analyses were performed using the standardized mean difference (SMD) under random‐ or fixed‐effects models according to heterogeneity levels.

**Results:**

Thirty‐four studies (1272 participants) were included (32.4% had a high risk of bias). Compared with controls, acute WBV showed no significant effect on handgrip strength, with moderate‐certainty evidence (SMD = 0.09, *p* = 0.34). Chronic WBV produced a significant but small effect, supported by very low‐certainty evidence (SMD = 0.33, *p* = 0.01). Sensitivity analysis excluding high‐risk‐of‐bias studies rendered the chronic effect non‐significant (*p* = 0.07). Exploratory subgroup analyses of chronic interventions showed a significant interaction for body positioning (*p* = 0.02), with significant effects for direct hand contact (SMD = 0.76, *p* = 0.03) and static standing (SMD = 0.52, *p* = 0.03). Compared with resistance training, evidence was inconclusive and of very low certainty (SMD = −2.43, *p* = 0.25).

**Conclusion:**

Chronic WBV involving direct hand contact may be associated with more pronounced effects on handgrip strength; however, these findings are exploratory, supported by very low‐certainty evidence, and require confirmation by future trials.

## 1 Introduction

Handgrip strength has been widely used as a key metric for assessing overall physical fitness and functional capacity, reflecting the integrity of the neuromuscular system and the ability to generate force during daily or high‐performance activities. It has emerged as an important biomarker of health (Vaishya et al. 2024). Reduced handgrip strength is associated with poorer functional performance, greater risk of disability, increased mortality, and adverse clinical outcomes across a variety of health conditions, making it a simple yet robust measure for monitoring general physical condition and responsiveness to interventions (Soysal et al. 2021).

From a physiological perspective, neuromuscular function can be modulated by external mechanical stimuli such as mechanical vibration, which can activate muscle spindles and type Ia afferent pathways, eliciting the tonic vibration reflex and increasing motor unit recruitment (Martin and Park 1997). Although these stimuli are typically applied to proximal body segments, such as in whole‐body vibration (WBV), where they are transmitted from the plantar surface of the feet via vibrating platforms, the resulting neural responses can extend beyond the site of stimulation. In this context, these neural responses may potentially influence distal muscle groups, including those involved in handgrip strength, although the magnitude of the mechanical vibration is substantially attenuated along its transmission through the body (Spain et al. 2021; Tankisheva et al. 2013).

Building on this physiological rationale, among the therapeutic and training strategies aimed at improving muscle strength, WBV therapy has gained prominence in recent decades (AlBaiti et al. 2024; Gonçalves de Oliveira et al. 2023; Qiu et al. 2025; Tan et al. 2023). WBV involves exposing the body to mechanical vibratory stimuli, which induces physiological changes such as increased muscle activation (AlBaiti et al. 2024). Previous studies have shown that WBV can enhance lower‐limb strength and power as well as functional performance in both clinical and healthy populations (Gonçalves de Oliveira et al. 2023; Qiu et al. 2025; Tan et al. 2023). However, its applicability for improving handgrip strength remains uncertain (Gonçalves de Oliveira et al. 2023).

Resistance training is widely recognized as the gold‐standard intervention for improving muscle strength, including that of the upper limbs, primarily through mechanisms involving voluntary muscle contractions, progressive overload, and neuromuscular adaptations. Evidence from recent systematic reviews and meta‐analyses indicates that resistance training can effectively improve handgrip strength across different populations, particularly in older adults and clinical groups with sarcopenia (Cheng et al. 2024; Hua‐Rui et al. 2025). In contrast, WBV induces muscle activation predominantly via reflex‐mediated neuromuscular responses and externally applied mechanical stimuli, without necessarily requiring targeted loading of specific muscle groups (Wang et al. 2022). Although both approaches may enhance neuromuscular function, their underlying mechanisms and modes of application differ substantially. To date, however, there is limited and inconsistent evidence directly comparing the effects of WBV and resistance training on handgrip strength, highlighting an important gap in the literature.

WBV interventions can be broadly categorised as acute (single‐session exposure) or chronic (repeated sessions over time), and these approaches are associated with distinct physiological responses. Acute WBV primarily elicits transient neuromuscular effects, such as increased muscle activation mediated by reflex pathways, including the tonic vibration reflex (Kalaoglu et al. 2023). In contrast, repeated sessions of WBV may promote cumulative neuromuscular adaptations that contribute to gains in muscle strength, primarily through neural mechanisms such as sustained increases in motor unit recruitment and improvements in neuromuscular coordination (Gonçalves de Oliveira et al. 2023; Wang et al. 2022) as well as potential morphological adaptations including muscle hypertrophy (Tian et al. 2025). These differences suggest that the duration of WBV exposure is a key factor influencing the magnitude and persistence of neuromuscular adaptations.

Therefore, the magnitude of WBV effects depends on multiple experimental and individual factors. These include participant characteristics (healthy individuals or those under healthcare management) (AlBaiti et al. 2024; Stania et al. 2016), intervention duration (acute vs. chronic protocols), and vibration parameters such as frequency and amplitude (Dolny and Reyes 2008). Moreover, body position during vibration exposure is a critical determinant of stimulus efficiency. The most common posture—standing with both feet on the platform—facilitates vibration transmission to the lower limbs, but progressive attenuation along the body substantially reduces the mechanical energy reaching the upper limbs (Spain et al. 2021). This dissipation raises questions about WBV's ability to induce meaningful adaptations in the muscles responsible for handgrip strength.

In contrast, positions involving direct hand contact with the vibration source—either directly on the platform or through attached handles—have been proposed as an alternative to enhance the stimulus magnitude in upper‐limb muscles. However, findings remain heterogeneous and inconsistent, varying according to exposure duration, vibration parameters, and study populations (Ahn et al. 2019; Coelho‐Oliveira et al. 2021; A. L. C. Souza et al. 2020; Dang and Chen 2019; Di Giminiani et al. 2014; Moreira‐Marconi et al. 2019; Goudarzian et al. 2017; Kurt and Pekünlü 2015; Lee et al. 2016; Morel et al. 2018; Pouyafar et al. 2021; Santos et al. 2020; Su and Chang 2024).

In the present review, direct hand contact with the vibration source is operationally defined as any body position in which the upper limbs are in physical contact with the vibrating stimulus during WBV exposure. This includes: (a) a push‐up position with the hands placed directly on the vibrating platform surface (Coelho‐Oliveira et al. 2021; A. L. C. Souza et al. 2020; Di Giminiani et al. 2014; Goudarzian et al. 2017; Morel et al. 2018; Pouyafar et al. 2021; Santos et al. 2020); (b) a seated position with the hands resting on the platform (Ahn et al. 2019; Dang and Chen 2019; Kurt and Pekünlü 2015; Lee et al. 2016); and (c) a standing or seated position with the hands gripping handles mechanically attached to the vibrating platform (García‐Gutiérrez et al. 2014; Su and Chang 2024). These configurations are distinguished from conventional standing protocols—whether static or with lower‐limb exercises—in which vibration is transmitted exclusively through the plantar surface of the feet, as they allow the mechanical vibration stimulus to be transmitted more directly to the upper limbs and the musculature involved in handgrip strength.

Given these methodological and physiological uncertainties, it is essential to critically synthesise the available evidence on the effects of WBV on handgrip strength. Therefore, the present study aimed to conduct a systematic review and meta‐analysis of randomized controlled trials (RCTs) to evaluate the impact of WBV on handgrip strength and to investigate potential moderators related to participant characteristics, intervention duration, vibration parameters, and the type of body contact with the vibration stimulus.

Based on the physiological rationale outlined above, we hypothesised that WBV would produce meaningful improvements in handgrip strength, and that protocols involving direct hand contact with the vibration source combined with chronic exposure would yield the greatest effects, given the reduced attenuation of the mechanical stimulus in the upper limbs and the potential for cumulative neuromuscular adaptations with repeated sessions.

## 2 Methods

This systematic review and meta‐analysis was prospectively registered in PROSPERO (CRD420251141715) and was conducted in accordance with the PRISMA 2020 statement (Page et al. 2021). Methodological procedures followed the recommendations outlined in the Cochrane Handbook for Systematic Reviews of Interventions (Higgins et al. 2023).

### 2.1 Eligibility Criteria

The inclusion criteria were as follows: (a) adult participants; (b) intervention involving whole‐body vibration (WBV); (c) at least one comparison group consisting of inactive controls, sham vibration, or other interventions aimed at improving muscle strength; (d) assessment of handgrip strength via a validated instrument; and (e) randomized study design. Health condition, WBV parameters, and body position on the vibration platform were not limited. Accordingly, subgroup analyses were performed to examine the effects of these potential moderators. Studies involving both acute (single‐session exposure) and chronic (repeated sessions over time) WBV interventions were included; however, these were analysed separately in independent main meta‐analyses.

### 2.2 Databases and Search Strategy

A systematic search was performed in the following databases: PubMed, Embase, CENTRAL, CINAHL, SPORTDiscus, Web of Science, LILACS, PEDro, and SciELO. The literature search was conducted from database inception up to September 5, 2025. No language restrictions were applied during the study selection process. To identify additional studies, the reference lists of the included trials and related systematic reviews were screened, and two clinical trial registries were consulted (https://clinicaltrials.gov/and https://trialsearch.who.int/). The search strategy was structured using terms related to the intervention (whole‐body vibration), outcome (handgrip strength), and study design (randomized clinical trial). Boolean operators OR were used to separate synonyms, and AND was used to combine term sets. The full search strategy for each database is provided in the Supporting Information S1.

### 2.3 Study Selection

Following the initial database search, all the references were imported into an online software platform (https://www.rayyan.ai/), where duplicates were automatically identified and manually verified. Two reviewers independently and blindly screened titles and abstracts to exclude studies that did not meet the inclusion criteria. The same reviewers then independently and blindly assessed the full texts of potentially eligible studies. Disagreements were resolved through discussion or consultation with a third reviewer.

### 2.4 Data Extraction

Two reviewers independently and blindly extracted data from each included study via a standardised form. Any discrepancies were resolved by a third reviewer. The extracted information included the following: (a) first author, publication year, and country; (b) number of participants, sex, health condition, and mean age per group; (c) duration and frequency of the interventions; (d) WBV protocol; (e) description of other interventions; (f) activities performed by inactive or sham‐vibration control groups; (g) instrument used to assess handgrip strength; (h) between‐group postintervention differences (mean, standard deviation, and statistical significance); (i) adverse events; and (j) adherence rate and session attendance frequency (compliance).

### 2.5 Risk of Bias Assessment

The risk of bias for each included study was assessed using the PEDro scale (Physiotherapy Evidence Database) (Maher et al. 2003). Two reviewers independently and blindly evaluated all the studies, and disagreements were resolved by a third reviewer. The PEDro scale comprises 11 items assessing internal validity and statistical reporting quality; the first item (related to external validity) is not scored. Thus, each randomized controlled trial (RCT) can obtain a maximum score of 10 points. Studies scoring < 6 points were classified as having a high risk of bias. Inter‐rater agreement for the PEDro scale assessments, calculated retrospectively via Cohen's Kappa, ranged from 0.60 to 0.79 across items, indicating substantial agreement (Landis and Koch 1977).

### 2.6 Data Analysis

For the meta‐analysis calculations, the effect measure was the postintervention standardised mean difference (SMD) between the WBV and control groups (inactive or sham vibration) or between the WBV and resistance training groups (traditional muscle‐strengthening exercises), accompanied by 95% confidence intervals (95% CI). Handgrip strength was reported in different units across studies (e.g., kgf, N, lbf). Since the SMD is a dimensionless statistic derived from within‐study variability, no unit conversion was required prior to pooling. Separate main meta‐analyses were conducted for acute and chronic WBV interventions for all primary comparisons. Cochrane's Q test was used to assess heterogeneity, which was considered significant when *p* ≤ 0.10. Heterogeneity was also quantified via the I^2^ statistic, interpreted as follows: 0%–40% (might not be important), 30%–60% (moderate heterogeneity), 50%–90% (substantial heterogeneity), and 75%–100% (considerable heterogeneity) (Higgins et al. 2023). Fixed effects models were applied when heterogeneity was not statistically significant (Cochrane's Q test *p* > 0.10 and I^2^ < 30%); otherwise, random effects models were used.

WBV effects on handgrip strength were considered statistically significant when *p* < 0.05. Effect sizes were interpreted as small (< 0.50), moderate (0.50–0.79), or large (≥ 0.80) (Cohen 1988). Publication bias was evaluated visually via funnel plots when ≥ 10 studies were available within the same analysis. Additionally, Egger's regression test was applied to assess funnel plot asymmetry statistically, with *p* < 0.05 indicating potential publication bias. All analyses were performed via Review Manager (RevMan, version 5.4; The Nordic Cochrane Centre, The Cochrane Collaboration, Copenhagen, Denmark) except for Egger's test, which was conducted via Jamovi software (version 2.6; The Jamovi Project, Sydney, Australia) via the MAJOR (Meta‐Analysis Jamovi Open Resource) module.

### 2.7 Sensitivity and Subgroup Analyses

Sensitivity analyses were conducted to determine whether the main findings remained consistent after excluding studies with a high risk of bias. Subgroup analyses were performed considering (a) health status (healthy participants vs. participants under healthcare management); (b) WBV intensity (high‐frequency [> 20 Hz] vs. low‐frequency vibration [≤ 20 Hz]), a threshold based on evidence that frequencies below 20 Hz induce muscular relaxation rather than neuromuscular training effects, whereas frequencies of 20–45 Hz are consistently associated with positive muscle strength outcomes (Rittweger 2010); and (c) body position (static standing, dynamic standing with exercise, or direct hand contact with the vibration source [hands placed directly on the platform or holding attached handles]). The sensitivity analyses were pre‐specified in the PROSPERO registration. All subgroup analyses, however, were conducted as exploratory analyses and were not pre‐specified.

The presence of a moderating effect was defined solely by the χ^2^ test for subgroup differences (*p* < 0.05). Significant within‐group effects in the absence of a statistically significant interaction test should be interpreted as exploratory observations only.

### 2.8 Certainty of Evidence

The certainty of the evidence was assessed via the GRADE approach (Grading of Recommendations, Assessment, Development, and Evaluation) (Schünemann et al. 2013). Two reviewers independently and blindly rated the evidence, and a third reviewer resolved disagreements. The GRADE framework includes domains that may lower the certainty of evidence: (a) risk of bias; (b) inconsistency; (c) indirectness; (d) imprecision; and (e) other factors (e.g., publication bias). After assessment, the certainty of evidence was categorised as follows: (a) High—further research is very unlikely to change the confidence in the effect estimate; (b) Moderate—further research is likely to have an important impact on the confidence in the estimate and may change it; (c) Low—further research is very likely to have an important impact on the estimate and likely to change it; or (1d) Very low—the effect estimate is highly uncertain.

## 3 Results

### 3.1 Identification and Screening of Studies

**FIGURE 1:**
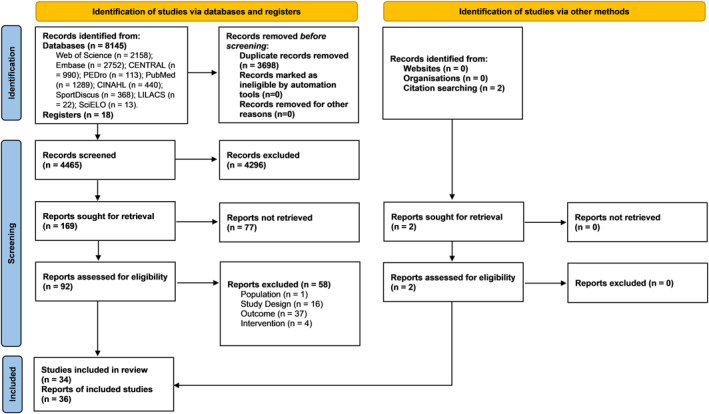
PRISMA flow diagram.

Figure 1 presents the PRISMA flow diagram. The database search yielded a total of 8163 records, including 8145 retrieved from electronic databases and 18 from clinical trial registries. After removing duplicates, 4465 records remained for title and abstract screening, 169 of which were selected for full‐text review. Among these, 77 records could not be retrieved—mainly clinical trial registrations without available results or conference abstracts (details of these unretrieved records are provided in Supporting Information S1: Table S1). Consequently, 92 full‐text reports were assessed for eligibility, and 58 were excluded for not meeting the inclusion criteria (a complete list with reasons for exclusion is available in Supporting Information S1: Table S2).

The reference lists of the 34 reports that met the inclusion criteria were screened, leading to the identification of two additional eligible reports. In total, 36 reports were included in the systematic review. However, two of these corresponded to duplicate publications from the same studies: Morel (2017) and Morel et al. (2018), as well as Torvinen et al. (2002), (2003), resulting in the inclusion of 34 unique studies in the systematic review. Of these, 32 provided sufficient data to be included in the meta‐analysis.

### 3.2 Characteristics of the Included Studies

Table 1 summarises the characteristics of the 34 studies included in the systematic review. The publication years ranged from 2002 to 2005, and studies were conducted in European (38.2%), Asian (35.3%), and South American (26.5%) countries. The total sample comprised 1272 participants, with most studies including both sexes (52.9%), while 26.5% included only women and 20.6% only men. With respect to health status, 18 studies included healthy participants, whereas 16 studies involved participants receiving healthcare or rehabilitation. WBV was most commonly compared with sham vibration (16 studies), inactive controls (14 studies), and other interventions (10 studies). Most studies (67.6%) implemented chronic (long‐term) interventions (mean duration: 11.2 ± 8.5 weeks), whereas the remainder used acute (single‐session) protocols. In long‐term studies, the mean training frequency was 3.3 ± 1.0 sessions per week, with an average session duration of 18.7 ± 9.5 min.

**TABLE 1:** Summary of studies included in the systematic review.

Study	Participants	Duration and frequency of interventions	WBV protocols	Other intervention protocols	Inactive control groups	Assessment of handgrip strength	Differences between groups	Adverse events (n)	Percentage of adherence and compliance^a^
Zhuang et al. (2025) China	27 older adults with sarcopenia: 13(M); 14(F) WBV = 14 (74 ± 4 y) RT = 13 (73 ± 4 y)	12 weeks 3x week 20 min	Vibration type: synchronous Frequency/amplitude: 12 Hz/4 mm Body positioning: Standing (knees at 30°) Exposure time: 10 sets (1 × 1 min rest)	RT (theraband): 7 exercises for upper and lower limbs Sets x rep.: 3 × 10	—	Jamar dynamometer	No difference	No adverse event	Adherence: 100% Compliance: WBV: 96% RT: 93%
Wong et al. (2025) China	62 older adults posthip surgery: 17(M); 45(F) WBV = 30 (82 ± 6 y) SHAM = 32 (84 ± 7 y)	14 days 5x week 20 min	Vibration type: NR Frequency/magnitude: 35 Hz/0.3 g Body positioning: Standing Exposure time: 20 min continuous	—	SHAM (false vibration)	Smedley dynamometer	No difference	WBV: Dizziness (3), foot pain (2), stomach pain (1) SHAM: Foot oedema (1)	Adherence: 97% Compliance: WBV: 87% SHAM: 84%
Cunha et al. (2025) Brazil	20 adults post prolonged covid‐19: 8(M); 12(F) WBV 2 mm = 7 (58 ± 8 y) WBV 4 mm = 7 (58 ± 8 y) SHAM = 6 (58 ± 7 y)	12 weeks 3x week 20 min	Vibration type: synchronous Frequency/amplitude: 35 Hz/2 or 4 mm Body positioning: Standing (knees at 15°) Exposure time: 13 sets (1 × 0.5 min rest)	—	SHAM (false vibration)	Smedley dynamometer	No difference	NR	Adherence: 77% Compliance: WBV 2 mm: 100% WBV 4 mm: 72% SHAM: 79%
Timón et al. (2024) Spain	52 healthy older adults: 24(M); 28(F) WBV normoxia = 13 (70 ± 4 y) WBV hypoxia = 12 (70 ± 5 y) Normoxia = 14 (71 ± 4 y) Hypoxia = 13 (70 ± 3 y)	20 weeks 3x week 20 min	Vibration type: side‐alternating Frequency/amplitude: 18–22 Hz/4 mm Body positioning: 4 exercises for lower and upper limbs with body weight and theraband Exposure time: 20 min continuous	—	Reading and using mobile apps	Smedley dynamometer	No difference	No adverse event	Adherence: 87% Compliance: 83%
Su and Chang (2024) Taiwan	58 institutionalised older adults with dynapenia: 26(M); 32(F) WBV = 29 (70 ± 4 y) CON = 29 (70 ± 5 y)	3 months 3x week 15 min	Vibration type: NR Frequency/amplitude: 25–40 Hz/2.5–5 mm Body positioning: Sitting on the platform, hands on the vibrating handle of the platform (arm vibration—AV) Exposure time: 10 sets (1 × 0.5 min rest)	—	Usual care	Charder dynamometer	WBV ↑ vs. CON	No adverse event	Adherence: NR Compliance: NR
H. C. M. Souza et al. (2022) Brazil	42 prefrail older women: 42(F) WBV + IMT = 14 (69 ± 4 y) WBV + IMTsham = 14 (69 ± 5 y) WBVsham + IMTsham = 14 (68 ± 4 y)	12 weeks WBV (3x week)/IMT (7x week) 10–30 min	Vibration type: synchronous Frequency/amplitude: 35 Hz/2–4 mm Body positioning: Standing (knees at 15°) Exposure time: 7‐20 sets (1 × 0.5 min rest)	IMT (inspiratory muscle training) 60 rep./day 40% MIP	WBVsham (false vibration) IMTsham (false IMT)	Smedley dynamometer	No difference	WBV: Itching (25), cramps (2) and dyspnoea (2) IMT: Nausea (3)	Adherence: 90% Compliance: NR
Sire et al. (2021) Italy	22 breast cancer patients: 11(F) WBV + exercise = 11 (52 ± 11 y) WBVsham + exercise = 11 (59 ± 10 y)	4 weeks 3x week 10 min	Vibration type: side‐alternating Frequency/amplitude: 30 Hz/1 mm Body positioning: Standing (knees at 30°) Exposure time: 5 sets (0.5 × 0.5 min rest)	RT: Squat exercises Sets x rep.: 5 × 10 Borg[10]: 4–6	SHAM (false vibration)	Jamar dynamometer	No difference	WBV + exercise: Nausea (1)	Adherence: 100% Compliance: NR
Santos et al. (2020) Brazil	19 healthy women: 19(F) (crossover) WBV push‐up = 19 (23 ± 4 y) WBVsham push‐up = 19 (23 ± 4 y) WBV standing = 19 (23 ± 4 y) WBVsham standing = 19 (23 ± 4 y) CON = 19 (23 ± 4 y)	1 session 5 min	Vibration type: synchronous Frequency/amplitude: 45 Hz/2 mm Body positioning: Push‐up (elbows at 10°) or standing (knees at 60°) Exposure time: 5 min continuous	—	CON (rest in seating position) or SHAM (false vibration)	Jamar dynamometer	WBV push‐up ↑ vs. WBV standing	NR	Not applicable (acute intervention study)
Pouyafar et al. (2021) Iran	34 healthy older men: 34(M) (All: 65 ± 4 y) WBV 40 Hz + jump rope = 12 WBV 25 Hz + jump rope = 12 CON = 10	8 weeks 3x week 30 min	Vibration type: NR Frequency/amplitude: 40 or 25 Hz/3 mm Body positioning: Push‐up and standing Exposure time: 4‐5 sets (1 × 0.5–2 min rest)	Jump rope: 30–35 jumps per min 2‐6 sets x 1 min Borg: 13–14	Usual routine	Jamar dynamometer	WBV 40 Hz and WBV 25 Hz ↑ vs. CON	NR	NR
Jo et al. (2021) Korea	40 healthy older adults: 18(M); 22(F) WBV + RT = 20 (74 ± 4 y) Stretching + RT = 20 (74 ± 5 y)	4 weeks 3x week 20 min	Vibration type: synchronous Frequency/amplitude: 10 Hz/5 mm Body positioning: Standing Exposure time: 20 min continuous	RT: 4 bodyweight exercises for upper and lower limbs Stretches: 10 exercises	—	Jamar dynamometer	No difference	No adverse event	Adherence: 100% Compliance: NR
Genest et al. (2021) Germany	47 older men with osteoporosis: 47(M) WBV = 13 (78 ± 6 y) RT = 11 (76 ± 6 y) Qi Gong = 10 (77 ± 8 y) Spinal orthosis = 13 (77 ± 6 y)	6 months 2x week 20–45 min	Vibration type: side‐alternating Frequency/amplitude: 5–25.5 Hz/0–2.5 mm Body positioning: Lower limb and trunk exercises Exposure time: 4‐7 sets (0.5–1 x 0.5–1 min rest)	RT: 8 exercises on equipment Qi Gong: Low‐impact movements and meditation	Spinal orthosis: Use of lumbar support for 3 h/day	DynEx1 dynamometer	No difference	No adverse event	Adherence: 100% Compliance: WBV: 83% RT: 71% Qi Gong: 65% Spinal orthosis: 85%
Coelho‐Oliveira et al. (2021) Brazil	21 women with rheumatoid arthritis: 21(F) (crossover) WBV push‐up = 21 (54 ± 11 y) WBVsham push‐up = 21 (54 ± 11 y) CON = 21 (54 ± 11 y)	1 session 5 min	Vibration type: synchronous Frequency/amplitude: 45 Hz/2 mm Body positioning: Push‐up (elbows at 10°) Exposure time: 5 min continuous	—	CON (rest in seating position) or SHAM (false vibration)	Jamar dynamometer	No difference	NR	Not applicable (acute intervention study)
A. L. C. Souza et al. (2020) Brazil	28 healthy women: 28(F) (crossover) WBV push‐up 25 Hz = 28 (27 ± 8 y) WBV push‐up 45 Hz = 28 (27 ± 8 y) WBVsham push‐up = 28 (27 ± 8 y) CON = 28 (27 ± 8 y)	1 session 5 min	Vibration type: synchronous Frequency/amplitude: 25 or 45 Hz/2 mm Body positioning: Push‐up (elbows at 10°) Exposure time: 5 min continuous	—	CON (rest in seating position) or SHAM (false vibration)	Jamar dynamometer	WBV 45 Hz ↑ vs. WBV 25 Hz, WBVsham and CON	No adverse event	Not applicable (acute intervention study)
Jepsen et al. (2020) Denmark	33 postmenopausal women with osteoporosis: 33(F) WBV + teriparatide = 15 (69 ± 5 y) Teriparatide = 18 (69 ± 8 y)	12 months 3x week 12 min	Vibration type: synchronous Frequency/amplitude: 30 Hz/1 mm Body positioning: Standing Exposure time: 6 sets (1 × 1 min rest)	—	Teriparatide: 20 μg s.c./day	Smedley dynamometer	No difference	WBV + teriparatide: Lower limb pain (2)	Adherence: 91% Compliance: WBV + teriparatide: 75% Teriparatide: 80%
Delkhoush et al. (2020) Iran	28 healthy adults: 14(M); 14(F) (crossover) WBV = 28 (23 ± 3 y) WBVsham = 28 (23 ± 3 y)	1 session 3 min	Vibration type: synchronous Frequency/amplitude: 35 Hz/2.5 mm Body positioning: Standing (knees at 30°) Exposure time: 3 min continuous	—	SHAM (false vibration)	Pneumatic dynamometer	No difference	No adverse event	Not applicable (acute intervention study)
Zhu et al. (2019) China	79 older sarcopenic men: 79(M) WBV = 28 (90 ± 4 y) Tai Chi = 24 (89 ± 4 y) CON = 27 (88 ± 3 y)	8 weeks 5x week 20 min	Vibration type: NR Frequency/amplitude: 12–16 Hz/3–5 mm Body positioning: Standing Exposure time: 5 sets (3 × 1 min rest)	Tai Chi: 10 movements (2 sets)	Usual routine	Jamar dynamometer	WBV ↑ vs. Tai Chi and CON	NR	Adherence: 86% Compliance: NR
Moreira‐Marconi et al. (2019) Brazil	94 adults with knee osteoarthritis: 79(M) WBV = 18 (NR y) Auriculotherapy = 19 (NR y) WBV + auriculotherapy = 16 (NR y) CON = 51 (NR y)	1 session and 5 weeks (2x week) 40 min	Vibration type: side‐alternating Frequency/amplitude: 5–14 Hz/2.5–7.5 mm Body positioning: Sitting on a chair with your feet on the platform Exposure time: 10 sets (3 × 1 min rest)	Auriculotherapy (ears stimulated with seeds)	NR	EMG system dynamometer	No difference	NR	Adherence: 84% Compliance: NR
Dang and Chen (2019) China	95 patients with hemiplegia after stroke: 60(M); 35(F) WBV = 32 (59 ± 9 y) WBV + TRT = 32 (61 ± 8 y) CON = 30 (60 ± 9 y)	4 weeks 5x week 15–30 min	Vibration type: NR Frequency/amplitude: 5–15 Hz/1–6 mm Body positioning: Sitting on a chair with his hands on the platform Exposure time: 15–30 min continuous	TRT: Task‐related training	Routine upper limb training (offered to the three groups)	Jamar dynamometer	WBV and WBV + task‐oriented ↑ vs. CON	NR	Adherence: 88% Compliance: NR
Ahn et al. (2019) Korea	60 patients with subacute stroke: 33(M); 27(F) WBV = 30 (59 ± 7 y) ULC = 30 (61 ± 6 y)	4 weeks 5x week 30 min	Vibration type: side‐alternating Frequency/amplitude: 4–19 Hz/1–6 mm Body positioning: Sitting on a chair with his hands on the platform Exposure time: 7 sets (2 × 2 min rest)	ULC: Upper‐ and lower‐cycle training	—	Jamar dynamometer	WBV ↑ vs. ULC	NR	Adherence: 100% Compliance: NR
Pessoa et al. (2018) Brazil	31 healthy older adults: 17(M); 14(F) WBV = 10 (66 ± 3 y) RT + WBVsham = 10 (68 ± 2 y) WBV + RT = 11 (65 ± 3 y)	12 weeks 3x week 40 min	Vibration type: synchronous Frequency/amplitude: 35 Hz/2–4 mm Body positioning: Standing (knees at 15°) Exposure time: 10‐20 sets (1 × 0.5 min rest)	RT: Exercises for upper and lower limbs	SHAM (false vibration)	Jamar dynamometer	No difference	No adverse event	Adherence: 91% Compliance: NR
Morel (2017); Morel et al. (2018) Brazil	15 healthy adult men: 15(M) (crossover) WBV standing = 15 (20 ± 2 y) WBV push‐up = 15 (20 ± 2 y) CON = 15 (20 ± 2 y)	1 session 30 s	Vibration type: synchronous Frequency/amplitude: 50 Hz/1.5 mm Body positioning: Standing (knees at 50°) or push‐up (elbows at 10°) Exposure time: 30 s continuous	—	CON (rest in seating position)	Jamar dynamometer	No difference	NR	Not applicable (acute intervention study)
Ebing et al. (2018) Germany	43 healthy adult men: 43(M) WBV synchronous = 14 (24 ± 4 y) WBV side‐alternating = 15 (26 ± 3 y) CON = 14 (25 ± 3 y)	10 weeks 2x week 10–20 min	Vibration type: synchronous or side‐alternating Frequency/amplitude: 24–30 Hz/4–5 mm Body positioning: Lower limb exercises Exposure time: 3‐4 sets (1–2 x 1 min rest)	—	7 upper body strength exercises (offered to the three groups)	Takei dynamometer	No difference	NR	Adherence: 100% Compliance: 100%
Goudarzian et al. (2017) Iran	22 healthy older women: 22(F) WBV + creatine = 8 (65 ± 3 y) WBV creatine placebo = 7 (66 ± 5 y) CON = 7 (68 ± 9 y)	10 days in a row 12 min	Vibration type: side‐alternating Frequency/amplitude: 30–35 Hz/5 mm Body positioning: Lower limb exercises and push‐up position Exposure time: 6 sets (1 × 1 min rest)	Creatine: 20 g/day (1st to 5th day) and 5 g/day (6th to 10th day)	Usual routine	Yagami dynamometer	No difference	No adverse event	Adherence: 100% Compliance: 98%
Lee et al. (2016) Korea	45 adults with poststroke hemiplegia: 24(M); 21(F) WBV = 15 (59 ± 8 y) WBV + TRT = 15 (59 ± 12 y) CON = 15 (60 ± 7 y)	4 weeks 3x week 21 min	Vibration type: side‐alternating Frequency/amplitude: 5–15 Hz/1–6 mm Body positioning: Sitting on a chair with his hands on the platform Exposure time: 7 sets (2 × 1 min rest)	TRT: Task‐related training	CON: Upper limb training, 1 h/day, 3x week WBV: Upper limb training, 30 min/day, 3x week	Jamar dynamometer	WBV + TRT ↑ vs. WBV and CON WBV ↑ vs. CON	NR	Adherence: 80% Compliance: NR
Santin‐Medeiros et al. (2015) Spain	37 healthy older women: 37(F) (All: 82 ± 6 y) WBV = 19 CON = 18	8 months 2x week 6 min	Vibration type: synchronous Frequency/amplitude: 20 Hz/2 mm Body positioning: Exercises for lower and upper limbs Exposure time: 6 sets (30 × 30 s rest)	—	Usual routine	Smedley dynamometer	No difference	NR	Adherence: 86% Compliance: NR
Kurt and Pekünlü (2015) Turkey	20 combat athletes: 8(M); 12(F) (crossover) WBV = 20 (23 ± 3 y) WBVsham = 20 (23 ± 3 y)	1 session 6 min	Vibration type: synchronous Frequency/amplitude: 26 Hz/4 mm Body positioning: Exercises with feet and hands/arms on the platform Exposure time: 4 sets (1 × 0.5 min rest)	—	SHAM (false vibration)	Takei dynamometer	WBV ↑ vs. WBVsham	NR	Not applicable (acute intervention study)
Casimiro et al. (2015) Brazil	21 healthy older women: 21(F) WBV = 10 (78 ± 4 y) CON = 11 (75 ± 3 y)	12 weeks 3x week 30 min	Vibration type: side‐alternating Frequency/amplitude: 35–40 Hz/2–4 mm Body positioning: Balance and strength exercises for the lower limbs Exposure time: 30 min continuous	—	Balance and strength exercises for the lower limbs (offered to both groups)	Jamar dynamometer	No difference	NR	Adherence: 88% Compliance: NR
Sievänen et al. (2014) Finland	13 institutionalised older adults with low physical function: 3(M); 10(F) WBV = 7 (84 ± 6 y) WBVsham = 6 (84 ± 9 y)	10 weeks 2x week 2–10 min	Vibration type: side‐alternating Frequency/amplitude: 12–18 Hz/2–8 mm Body positioning: Lower limb exercises Exposure time: 1‐5 sets (1 × 1 min rest)	—	SHAM (false vibration)	Lafaytte dynamometer	No difference	NR	Adherence: 87% Compliance: WBV: 74% WBVsham: 73%
García‐Gutiérrez et al. (2014) Spain	28 healthy adult men: 15(M) (crossover) WBV AV = 28 (22 ± 2 y) AV = 28 (22 ± 2 y) WBVsham = 28 (22 ± 2 y)	1 session 40 s	Vibration type: synchronous Frequency/amplitude: 50 Hz/2.5 mm Body positioning: Standing (knees at 30°), hands on the vibrating handle of the platform (arm vibration—AV) Exposure time: 40 s continuous	—	SHAM (false vibration)	Takei dynamometer	No difference	NR	Not applicable (acute intervention study)
Di Giminiani et al. (2014) Italy	30 healthy adult men: 30(M) WBV 40 Hz = 10 (25 ± 1 y) WBV 20 Hz = 10 (26 ± 2 y) WBVsham = 10 (24 ± 1 y)	1 session 80 s	Vibration type: synchronous Frequency/amplitude: 40 Hz/0.9 mm or 20 Hz/0.2 mm Body positioning: Push‐up (elbows at 10°) Exposure time: 20 sets (10 × 10 s rest)	—	SHAM (false vibration)	Ergotest‐Innovation dynamometer	No difference	No adverse event	Not applicable (acute intervention study)
Bautmans et al. (2005) Belgium	21 institutionalised older adults with low physical function: 8(M); 13(F) WBV = 10 (76 ± 10 y) WBVsham = 11 (79 ± 10 y)	6 weeks 3x week 5–10 min	Vibration type: synchronous Frequency/amplitude: 35–40 Hz/2–5 mm Body positioning: Lower limb exercises Exposure time: 1‐3 sets (0.5–1 x 0.5–1 min rest)	—	SHAM (false vibration)	Martin Vigorimeter dynamometer	No difference	WBV: Groin pain (1), airway infection (1)	Adherence: 88% Compliance: WBV: 96% WBVsham: 86%
Torvinen et al. (2002), (2003) Finland	53 healthy adults: 19(M); 34(F) WBV = 27 (23 ± 4 y) CON = 26 (26 ± 6 y)	8 months 3–5x week 4 min	Vibration type: NR Frequency/amplitude: 25–45 Hz/2 mm Body positioning: Standing exercises with weight transfer Exposure time: 4 min continuous	—	Usual routine	Digitest dynamometer	No difference	No adverse event	Adherence: 95% Compliance: 75%
Torvinen, Kannu, et al. (2002) Finland	16 healthy adults: 8(M); 8(F) (crossover) WBV = 16 (24–33 y) WBVsham = 16 (24–33 y)	1 session 4 min	Vibration type: side‐alternating Frequency/amplitude: 15–30 Hz/10 mm Body positioning: Standing exercises with weight transfer Exposure time: 4 min continuous	—	SHAM (false vibration)	Digitest dynamometer	No difference	No adverse event	Not applicable (acute intervention study)
Torvinen, Sievänen, et al. (2002) Finland	16 healthy adults: 8(M); 8(F) (crossover) WBV = 16 (18–35 y) WBVsham = 16 (18–35 y)	1 session 4 min	Vibration type: NR Frequency/amplitude: 25–40 Hz/2 mm Body positioning: Standing exercises with weight transfer Exposure time: 4 min continuous	—	SHAM (false vibration)	Digitest dynamometer	No difference	No adverse event	Not applicable (acute intervention study)

Abbreviations: CON, control; F, female; IMT, inspiratory muscle training; M, male; MIP, maximum inspiratory pressure; RT, resistance training; SHAM, simulated (fake) intervention; TRT, task‐related training; ULC, upper‐ and lower‐cycle training; WBV, whole‐body vibration; y, years old.

^a^ Adherence: percentage of participants who remained until the end of the intervention period; Compliance: percentage of participation/attendance during the intervention period.

WBV protocols involved synchronous vibration in 50% of the studies and side‐alternating vibration in 32.4%, whereas the remaining studies did not specify the vibration type. With respect to vibration parameters, most studies (64.7%) have used high‐frequency WBV (> 20 Hz), and the mean peak‐to‐peak displacement amplitude was 3.2 ± 1.8 mm. With respect to body position, participants most frequently performed WBV in a static standing posture with slightly flexed knees (32.4%) or while performing strengthening exercises primarily targeting the lower limbs (29.4%). The remaining 38.2% of studies used positions involving direct hand contact with the vibration stimulus (either hands placed directly on the vibration platform or holding handles attached to it). Most studies (94.1%) assessed handgrip strength via hydraulic dynamometers, whereas one study used a pneumatic dynamometer (Delkhoush et al. 2020), and another used a Vigorimeter (Bautmans et al. 2005).

When comparing groups, seven studies reported that WBV produced superior effects compared with inactive or sham controls (A. L. C. Souza et al. 2020; Dang and Chen 2019; Kurt and Pekünlü 2015; Lee et al. 2016; Pouyafar et al. 2021; Su and Chang 2024; Zhu et al. 2019). One study reported that WBV performed in a push‐up position with hands on the platform was superior to that performed in the standing position (Santos et al. 2020), and two studies reported that WBV outperformed other interventions (arm and leg cycling exercises (Ahn et al. 2019) and Tai Chi Chuan (Zhu et al. 2019)). The remaining studies reported no significant differences in handgrip strength.

Among the adverse events associated with WBV, 14.7% of the studies reported such events, affecting a total of 44 participants. All studies reporting adverse events employed high‐frequency vibration (30–40 Hz) with participants in a standing position or performing lower‐limb exercises; no adverse events were reported in studies involving direct hand contact with the vibration stimulus. The reported events were mild and transitory, including dizziness, nausea, foot pain, lower‐limb pain, stomach discomfort, groin pain, itching, cramps, respiratory infection, and dyspnoea. No serious adverse events were reported. In contrast, 38.2% of the studies reported no adverse events, and the remainder did not mention this outcome. Finally, intervention adherence ranged from 77% to 100%, and compliance rates ranged from 72% to 100%.

### 3.3 Risk of Bias of Included Studies

Among the 34 studies included in the systematic review, approximately one‐third (32.4%) presented a high risk of bias (PEDro score < 6). Across all the studies, the mean PEDro score was 6.0 ± 1.3 points, ranging from 3 to 9 points (Table 2).

**TABLE 2:** Risk of bias of studies assessed by the PEDro scale.

Study	C1^a^	C2	C3	C4	C5	C6	C7	C8	C9	C10	C11	Score^b^ (0–10)
Zhuang et al. (2025)	+	+	−	+	−	−	+	+	+	+	+	7
Wong et al. (2025)	+	+	+	−	+	−	+	+	+	+	+	8
Cunha et al. (2025)	+	+	+	−	+	−	−	−	−	+	+	5
Timón et al. (2024)	+	+	−	+	−	−	−	+	−	+	+	5
Su and Chang (2024)	+	+	−	+	−	−	−	−	−	+	+	4
H. C. M. Souza et al. (2022)	+	+	+	+	+	−	+	+	+	+	+	9
Sire et al. (2021)	+	+	−	+	+	−	+	+	+	+	+	8
Santos et al. (2020)	+	+	+	+	−	−	−	+	+	+	+	7
Pouyafar et al. (2021)	−	+	−	+	−	−	−	+	−	+	+	5
Jo et al. (2021)	+	+	−	+	−	−	+	+	+	+	+	7
Genest et al. (2021)	+	+	−	+	−	−	+	+	+	−	+	6
Coelho‐Oliveira et al. (2021)	+	+	−	+	−	−	−	+	+	+	+	6
A. L. C. Souza et al. (2020)	−	+	−	+	−	−	−	+	+	+	+	6
Jepsen et al. (2020)	+	+	−	+	−	−	+	+	+	+	−	6
Delkhoush et al. (2020)	+	+	−	+	−	−	−	+	+	+	+	6
Zhu et al. (2019)	+	+	−	+	−	−	−	+	−	+	+	5
Moreira‐Marconi et al. (2019)	+	+	−	−	−	−	−	−	−	+	+	3
Dang and Chen (2019)	+	+	−	+	−	−	−	+	−	+	+	5
Ahn et al. (2019)	+	+	−	+	−	−	−	+	+	+	+	5
Pessoa et al. (2018)	−	+	+	+	−	−	+	+	−	+	+	7
Morel (2017); Morel et al. (2018)	+	+	−	+	−	−	−	+	+	+	+	6
Ebing et al. (2018)	+	+	−	+	−	−	−	+	+	+	+	6
Goudarzian et al. (2017)	+	+	−	+	−	−	+	+	+	+	+	7
Lee et al. (2016)	+	+	−	+	−	−	+	−	−	+	+	5
Santin‐Medeiros et al. (2015)	+	+	−	−	−	−	−	+	−	+	+	4
Kurt and Pekünlü (2015)	−	+	−	+	−	−	−	+	+	+	+	6
Casimiro et al. (2015)	+	+	−	+	−	−	+	+	−	+	+	6
Sievänen et al. (2014)	+	+	−	+	+	−	+	+	+	+	+	8
García‐Gutiérrez et al. (2014)	+	+	−	+	−	−	−	+	+	+	+	6
Di Giminiani et al. (2014)	+	+	−	+	+	−	+	+	+	+	+	8
Bautmans et al. (2005)	+	+	−	+	+	−	−	+	−	+	+	6
Torvinen et al. (2002), (2003)	+	+	−	+	−	−	−	+	−	+	+	5
Torvinen, Kannu, et al. (2002)	−	+	−	+	−	−	−	+	+	+	+	6
Torvinen, Sievänen, et al. (2002)	−	+	−	+	−	−	−	+	+	+	+	6

C1: Have the eligibility criteria been specified?

C2: Were the subjects randomly assigned to the groups?

C3: Was the placement of subjects blinded?

C4: Was there baseline comparability?

C5: Did all the subjects participate blindly in the study?

C6: Did all the therapists blindly administer the therapies?

C7: Did all the raters blindly measure at least one key outcome?

C8: Were measurements of at least one key outcome obtained in more than 85% of the subjects initially randomized?

C9: Was an intention‐to‐treat analysis performed?

C10: Were comparisons made between groups?

C11: Have point and variability estimates been presented?

^a^ Item not scored.

^b^ Overall average (standard deviation): 6.0 (1.3); +: criterion met; −: criterion not met.

### 3.4 Quantitative Synthesis

**FIGURE 2:**
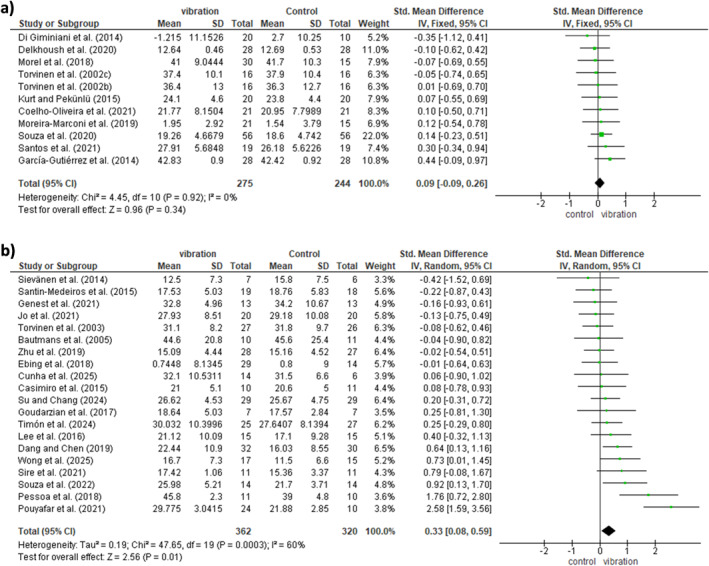
Meta‐analysis comparing whole‐body vibration therapy vs. control groups for handgrip strength: (a) acute interventions; (b) chronic interventions.

**FIGURE 3:**
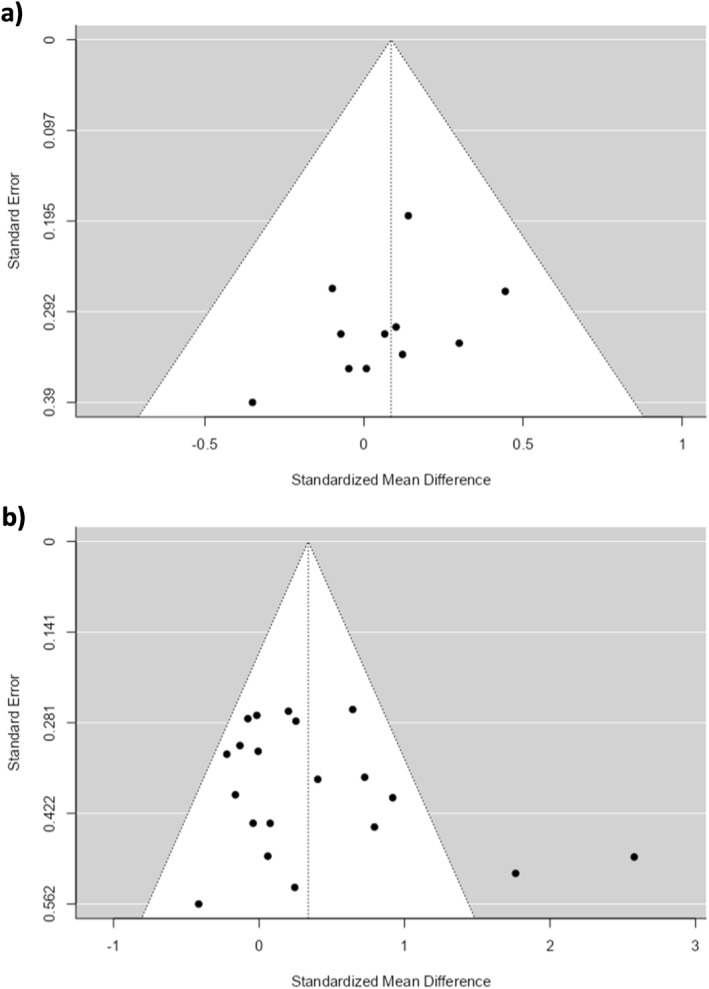
Funnel plot of the comparison between whole‐body vibration therapy vs. control groups for handgrip strength: (a) acute interventions; (b) chronic interventions.

**FIGURE 4:**
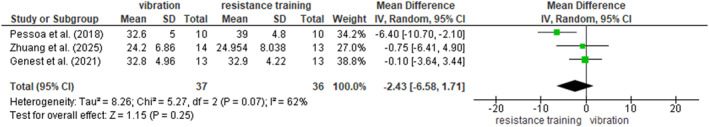
Meta‐analysis comparing whole‐body vibration therapy vs. resistance training for handgrip strength—chronic interventions.

Compared with inactive controls or sham vibration, acute WBV interventions produced no significant effect on handgrip strength, with moderate‐certainty evidence (downgraded due to indirectness) and no indication of publication bias (SMD = 0.09 [95% CI −0.09 to 0.26], I^2^ = 0%, *p* = 0.34, *n* = 519; Figure 2a; Egger's regression: intercept = −0.780; *p* = 0.435; Figure 3a). Chronic WBV interventions yielded a statistically significant but small effect (SMD = 0.33 [95% CI 0.08 to 0.59], I^2^ = 60%, *p* = 0.01, *n* = 682; Figure 2b), with a marginal indication of potential publication bias (Egger's regression: intercept = 1.587; *p* = 0.113; Figure 3b); however, the certainty of evidence was rated as very low (downgraded due to risk of bias, inconsistency, and indirectness), and by conventional benchmarks an SMD of 0.33, while statistically significant, remains small and likely represents a marginal improvement of limited clinical relevance in isolation. When WBV was compared with resistance training, very low‐certainty evidence (downgraded due to inconsistency, indirectness, and imprecision) did not allow any conclusion regarding potential differences between interventions (SMD = −2.43 [95% CI −6.58 to 1.71], I^2^ = 62%, *p* = 0.25, *n* = 73; Figure 4), as this estimate is based on only three small studies and is therefore highly unstable. Details of the certainty‐of‐evidence assessments are presented in Table 3.

**TABLE 3:** Analysis of the certainty of evidence via the GRADE system.

No of studies	Certainty assessment				№ of participants	SMD (95% CI)	Certainty of evidence
	Risk of bias	Inconsistency	Indirectness	Imprecision			
Whole body vibration therapy vs. control groups—acute interventions							
11	not serious	not serious	serious^a^	not serious	519	0.09 (−0.09, 0.26)	⨁⨁⨁◯ moderate^a^
Whole body vibration therapy vs. control groups—chronic interventions							
20	serious^b^	serious^c^	serious^a^	not serious	682	0.33 (0.08, 0.59)	⨁◯◯◯ very low^a^ ^,^ ^b^ ^,^ ^c^
Whole body vibration therapy vs. resistance training—chronic interventions							
3	not serious	serious^c^	serious^a^	serious^d^	73	−2.43 (−6.58, 1.71)	⨁◯◯◯ very low^a^ ^,^ ^b^ ^,^ ^c^

Abbreviations: 95% CI, 95% confidence interval; SMD, standardised mean difference.

^a^ Heterogeneous population, involving participants with different health conditions.

^b^ Statistical significance was lost when only studies at low risk of bias were included.

^c^ There was high heterogeneity among the studies.

^d^ The sample size was below the optimal information size.

#### 3.4.1 Sensitivity Analyses

Sensitivity analyses were conducted separately for acute and chronic interventions to assess the influence of studies with a high risk of bias. After excluding these studies, the result for acute interventions remained non‐significant (SMD = 0.08 [95% CI −0.10 to 0.26], I^2^ = 0%, *p* = 0.37, *n* = 483; Supporting Information S1: Figure S1a). For chronic interventions, the exclusion of high‐risk‐of‐bias studies rendered the previously significant result non‐significant (SMD = 0.32 [95% CI −0.03 to 0.66], I^2^ = 48%, *p* = 0.07, *n* = 281; Supporting Information S1: Figure S1b), indicating that the main finding for chronic interventions should be interpreted with caution.

#### 3.4.2 Subgroup Analyses

The results of the subgroup analyses are presented in Table 4 and Supporting Information S1: S2 to S7. For acute interventions, no significant within‐group effects were observed in any subgroup, and no interaction test reached statistical significance. For chronic interventions, although the interaction test between health condition subgroups did not reach statistical significance (*p* = 0.72), a significant within‐group effect was observed among healthcare participants (SMD = 0.32 [95% CI 0.08–0.55], *p* = 0.008), whereas no significant effect was found among healthy participants (*p* = 0.11). Regarding vibration intensity in chronic interventions, a significant within‐group effect was observed in the high‐frequency subgroup (SMD = 0.55 [95% CI 0.14–0.96], *p* = 0.008) but not in the low‐frequency subgroup (*p* = 0.35); however, the interaction test between subgroups did not reach statistical significance (*p* = 0.07). Regarding body positioning in chronic interventions, significant within‐group effects were observed in the direct hand contact subgroup (SMD = 0.76 [95% CI 0.08–1.44], *p* = 0.03) and the static standing subgroup (SMD = 0.52 [95% CI 0.05–0.98], *p* = 0.03), but not in the dynamic standing subgroup (*p* = 0.80). Notably, the interaction test between body positioning subgroups reached statistical significance (*p* = 0.02), indicating a statistically significant difference between subgroups for this variable in chronic interventions.

**TABLE 4:** Statistics for subgroup analyses.

Subgroup	No of studies	No of participants	SMD (95% CI)	Within‐group		Between‐group	
				I^2^	*P*	I^2^	*P*
Whole body vibration therapy vs. control groups—acute interventions							
Health condition							
Healthy participants	9	441	0.08 (−0.11, 0.27)	0%	0.40		
Healthcare participants	2	99	0.13 (−0.28, 0.54)	0%	0.54	0%	0.84
Vibration intensity							
High frequency	10	856	0.09 (−0.09, 0.27)	0%	0.33		
Low frequency	01	36	0.12 (−0.54, 0.78)	—	0.72	0%	0.93
Body positioning							
Hands in contact with vibration	07	369	0.14 (−0.07, 0.35)	0%	0.19		
Standing (static position)	01	56	−0.10 (−0.62, 0.42)	—	0.71		
Standing (performing exercises)	02	64	−0.02 (−0.51, 0.47)	0%	0.94	0%	0.64
Whole body vibration therapy vs. control groups—chronic interventions							
Health condition							
Healthy participants	09	315	0.42 (−0.09, 0.92)	77%	0.11		
Healthcare participants	11	682	0.32 (0.08, 0.55)	17%	0.008	0%	0.72
Vibration intensity							
High frequency	12	367	0.55 (0.14, 0.96)	69%	0.008		
Low frequency	08	315	0.12 (−0.13, 0.36)	15%	0.35	69%	0.07
Body positioning							
Hands in contact with vibration	05	198	0.76 (0.08, 1.44)	78%	0.03		
Standing (static position)	07	218	0.52 (0.05, 0.98)	61%	0.03		
Standing (performing exercises)	08	266	−0.03 (−0.27, 0.21)	0%	0.80	73.8%	0.02

Abbreviations: 95% CI, 95% confidence interval; SMD, standardised mean difference.

## 4 Discussion

This study comprehensively synthesised the available evidence on the effects of WBV on handgrip strength. A total of 34 studies were included, 32 of which provided sufficient data for inclusion in the meta‐analysis. Acute WBV interventions produced no significant effect on handgrip strength, with moderate‐certainty evidence (SMD = 0.09). Chronic WBV interventions yielded a statistically significant but small effect, with very low‐certainty evidence (SMD = 0.33). Although statistically significant, this effect size remains small by conventional benchmarks and warrants critical appraisal in clinical terms. The Minimal Clinically Important Difference (MCID) for handgrip strength has been estimated at 5.0–6.5 kg (Bohannon 2019), and an SMD of 0.33, while statistically significant, is unlikely to reflect an absolute improvement reaching this threshold in most clinical groups. Therefore, the routine prescription of WBV solely for handgrip improvement — particularly considering the acquisition and maintenance costs of vibration platforms and the time investment required by frail patients — appears difficult to justify on the basis of the pooled evidence alone. More clinically meaningful effects may be achievable under specific conditions, as explored in the subgroup analyses. In contrast, no conclusion could be drawn from the comparison between WBV and resistance training, as this estimate is based on only three small studies with very low‐certainty evidence.

In the main comparison against control groups, chronic WBV interventions yielded a statistically significant but small effect (SMD = 0.33), while acute interventions produced no significant effect (SMD = 0.09). The chronic effect size was smaller than those reported in recent meta‐analyses investigating the effects of WBV on lower‐limb strength, where moderate effect sizes have typically been observed (SMD > 0.50) (Gonçalves de Oliveira et al. 2023; Qiu et al. 2025; Tan et al. 2023). Those meta‐analyses included only chronic interventions performed in a standing position, where vibration is primarily absorbed by the muscles and joints of the lower limbs, thereby promoting more pronounced neuromuscular responses in that region. In contrast, the present meta‐analysis specifically examined handgrip strength, which explains the smaller overall effect observed, as mechanical energy is largely dissipated through the lower limbs when participants stand on the vibration platform, resulting in limited stimulation of the upper segments (Spain et al. 2021).

Substantial clinical and methodological heterogeneity was observed across the included studies, involving differences in participant health status, WBV protocols, and outcome assessment methods. This heterogeneity was systematically explored through subgroup analyses. Although the interaction tests between subgroups did not reach statistical significance for most comparisons, significant within‐group effects were consistently observed among healthcare participants in high‐frequency protocols, and when body positioning involved direct hand contact with the vibration stimulus in chronic interventions. Notably, the interaction test for body positioning in chronic interventions was the only comparison to reach statistical significance (*p* = 0.02), driven by the absence of effect in the dynamic standing subgroup. These observations are exploratory and should not be interpreted as evidence of confirmed moderation; however, they may inform hypothesis generation for future trials. The sensitivity analysis excluding studies with high risk of bias rendered the main chronic effect non‐significant, reinforcing the very low certainty of evidence rating and highlighting the need for methodologically rigorous trials in this field.

Among chronic interventions, the subgroup analyses revealed moderate and statistically significant within‐group effects for two body positioning conditions: direct hand contact with the vibration stimulus (SMD = 0.76) and static standing (SMD = 0.52), whereas no significant effect was observed in the dynamic standing subgroup. The effect associated with direct hand contact is physiologically coherent, as configurations such as the push‐up position or gripping handles directly coupled to the vibrating platform place the hands, wrists, and forearms in immediate proximity to the vibration source, enabling direct mechanical stimulation of the relevant musculature. In the static standing condition, however, the substantial attenuation of vibratory wave amplitude across successive joint interfaces renders direct transmission to the upper limbs biomechanically implausible as the primary mechanism (Harazin and Grzesik 1998; Spain et al. 2021). A more parsimonious explanation is that the sustained isometric contractions demanded by the upper limbs while gripping the platform support to maintain postural stability during vibration exposure may themselves constitute an adequate neuromuscular stimulus for handgrip strength adaptation. Consistent with this interpretation, no significant effect was observed in the dynamic standing subgroup, where movement‐based exercise typically precludes reliance on upper‐limb gripping for postural control. Future studies incorporating concurrent accelerometry and surface electromyography at the forearm and hand are warranted to empirically disentangle these mechanisms.

Among chronic studies, the average intervention duration was 11.2 ± 8.5 weeks, with a mean training frequency of 3.3 ± 1.0 sessions per week and an average session duration of 18.7 ± 9.5 min. These parameters suggest that chronic WBV interventions enable the consolidation of neuromuscular adaptations, such as greater motor unit recruitment and synchronisation, improved coordination, and potential morphological changes in muscles and tendons (Keller et al. 2013; Łochyński et al. 2013). Conversely, a single acute session appears insufficient to trigger structural or functional adaptations in the forearm and hand muscles, supporting the notion that WBV follows similar training principles to traditional resistance exercise, relying on cumulative exposure and temporal consistency (Pearcey et al. 2021).

Another possible determinant of WBV effectiveness appears to be vibration intensity. In the present review, among chronic interventions, statistically significant within‐group improvements were observed exclusively in high‐frequency protocols (> 20 Hz), whereas low‐frequency vibration produced no significant impact. High‐frequency vibration enhances muscle spindle activation and intensifies the tonic vibration reflex, resulting in greater electromyographic activity and motor unit recruitment. In contrast, low‐frequency vibration transmits less acceleration to the muscles, producing limited activation of the spindles and, consequently, a weaker tonic vibration reflex—an essential mechanism for rapid and synchronised motor unit recruitment (AlBaiti et al. 2024). Depending on the body position, this effect may be further attenuated owing to mechanical dissipation of vibration energy as it propagates through the body. Thus, at low intensities, the vibratory energy likely fails to reach distal muscles with sufficient magnitude to induce functional overload (Harazin and Grzesik 1998).

While high‐frequency vibration combined with direct hand contact appears to optimise neuromuscular outcomes, this configuration warrants careful consideration of potential safety risks. Hand‐Arm Vibration Syndrome (HAVS) is a well‐documented occupational condition resulting from prolonged, repeated exposure of the upper limbs to mechanical vibration, characterised by vascular, neurological, and musculoskeletal impairment (ISO 5349–1:2001). Nonetheless, the WBV protocols examined in this review differ substantially from occupational exposure scenarios: sessions were of short duration (mean 18.7 ± 9.5 min), performed at limited weekly frequency (mean 3.3 sessions/week), and involved intermittent rather than continuous vibration. Furthermore, no adverse events attributable to upper‐limb vibration were reported in any of the hand‐contact studies included. While these observations are reassuring, the available trials lacked long‐term follow‐up and systematic monitoring of upper‐limb sensory and vascular function, precluding definitive conclusions regarding chronic safety. Future studies employing direct hand‐contact protocols should incorporate standardised assessment of upper‐limb neurovascular function to characterise the safety profile of this intervention configuration more rigorously.

Regarding the study populations, because the inclusion criteria did not restrict participant health status, subgroup analyses were conducted to examine potential differences between healthy individuals and those receiving healthcare. Among chronic interventions, a significant within‐group effect was observed exclusively among healthcare participants (SMD = 0.32), with no significant effect observed among healthy individuals (SMD = 0.42, *p* = 0.11). This differential responsiveness is consistent with a ceiling effect: individuals with lower baseline handgrip strength—such as those with sarcopenia, osteoporosis, or stroke sequelae—exhibit greater neuromuscular plasticity and are therefore more sensitive to vibratory stimuli. In contrast, healthy individuals, and particularly those who are physically active or athletically trained, operate closer to their physiological ceiling, which inherently limits the magnitude of any training‐induced gain (Caprioli et al. 2024). The low heterogeneity in the healthcare subgroup (I^2^ = 17%) supports the consistency of this effect across diverse clinical conditions. These findings suggest that baseline handgrip strength levels may be an important determinant of intervention responsiveness, and future trials should consider stratifying participants accordingly to better identify population profiles most likely to benefit from WBV.

Compared with resistance training, the present meta‐analysis revealed no significant differences; however, this comparison was based on only three studies involving a total of 73 participants, which considerably limits the strength of the evidence. The analysis included only three studies, preventing further subgroup exploration. These studies had small sample sizes and all used standing WBV protocols—either in a static position (Pessoa et al. 2018; Zhuang et al. 2025) or during exercise (Genest et al. 2021)—without direct hand contact with the vibration stimulus. This likely restricted the transmission of vibratory energy to the upper limbs and consequently the potential impact on handgrip strength. Given the very small number of studies and participants, these findings should be interpreted as inconclusive rather than indicative of equivalence between interventions.

A previous meta‐analysis demonstrated that conventional exercise, including resistance training, yielded a small but significant improvement in handgrip strength among healthy older adults (SMD = 0.28) (Labott et al. 2019). The mean intervention duration in those studies was 22 weeks, suggesting that to achieve greater improvements in handgrip strength, specific training targeting the forearm and hand muscles is likely required—which is consistent with the findings of our subgroup analyses.

Finally, WBV was generally well tolerated with a low incidence of adverse events (14.7% of studies). The reported adverse events included dizziness, nausea, foot or lower‐limb pain, abdominal or groin pain, itching, cramps, respiratory infection, and dyspnoea, affecting a total of 44 participants (3.5%) in this systematic review. No serious adverse events were reported, supporting the safety profile of WBV. Adherence to the intervention was high (77%–100%), with compliance ranging from 72% to 100%, indicating good acceptability even among clinical populations with functional limitations. This high adherence, combined with the low rate of adverse events, reinforces WBV's potential as a feasible and well‐tolerated intervention strategy, particularly for individuals under healthcare who may face difficulties engaging in conventional exercise programs.

### 4.1 Certainty of Evidence

The certainty of evidence was assessed via the GRADE approach, and the results ranged from very low to moderate certainty. As no outcome reached high‐certainty evidence, the emergence of new RCTs may alter the current confidence in the effect estimates—an important consideration for interpretation. For the comparison of WBV versus control groups, acute interventions were rated as moderate‐certainty evidence (downgraded due to indirectness), whereas chronic interventions were rated as very low‐certainty evidence (downgraded due to risk of bias, inconsistency, and indirectness). The comparison of WBV versus resistance training was downgraded owing to inconsistency, indirectness, and imprecision.

The downgrading of indirectness in the main analysis was due to the inclusion of both healthy participants and those under healthcare. It is difficult to assume that WBV would exert equivalent effects on handgrip strength across all health conditions. Nevertheless, we opted to pool these populations for three main reasons: (1) there is biological plausibility for a shared neuromuscular mechanism of vibratory stimulation (motor recruitment, tonic stretch reflex) across different clinical contexts; (2) such pooling increases statistical power and representativeness, providing a broader view of the intervention's impact; and (3) a more inclusive strategy minimises selection bias that could otherwise overestimate effects by excluding clinically relevant populations.

To mitigate potential bias from population mixing, subgroup analyses (healthy vs. under healthcare) were performed. For the subgroup under healthcare, despite distinct clinical conditions, heterogeneity remained low (I^2^ = 17%). Heterogeneity was absent in the acute model (I^2^ = 0%) and substantial in the chronic model (I^2^ = 60%), the latter contributing to the downgrading of certainty for chronic interventions.

The risk of bias was assessed via the PEDro scale, with fewer than one‐third of the studies (32.4%) classified as high risk (PEDro < 6) and a mean score of 6.0 ± 1.3 across the 34 studies, indicating an overall acceptable methodological quality. The main limitations were related to a lack of participant and therapist blinding, which is often challenging in perceptible interventions such as WBV. Notably, the sensitivity analysis excluding studies at high risk of bias confirmed the non‐significant result for acute interventions but rendered the chronic effect non‐significant (*p* = 0.07), suggesting that the significant pooled estimate for chronic interventions may be partly driven by studies with higher risk of bias, and reinforcing the very low certainty of evidence rating for this comparison.

This methodological limitation should be considered when interpreting the findings, as the absence of blinding may introduce performance and detection bias, potentially inflating the observed effects. Therefore, although the overall risk of bias was not considered serious in the GRADE assessment, the internal validity of some included studies may be partially compromised, warranting cautious interpretation of the pooled estimates.

Regarding inconsistency, substantial heterogeneity (I^2^ = 62%) was observed in the WBV versus resistance training comparison, likely reflecting fundamental design differences among the three included studies. These studies diverged across multiple dimensions: population clinical profile (healthy older adults, sarcopenic older adults, and older men with osteoporosis), vibration type and frequency (synchronous 35 Hz, synchronous 12 Hz, and side‐alternating 5–25.5 Hz), intervention duration (12 weeks, 12 weeks, and 6 months), weekly training frequency (3, 3, and 2 sessions/week), and the nature of the resistance training comparator (theraband‐based exercises vs. machine‐based progressive resistance training). Additionally, all three studies used standing WBV protocols without direct hand contact, which likely limited vibration transmission to the upper limbs. These heterogeneous characteristics preclude a homogeneous pooled estimate.

This small evidence base warrants larger, high‐quality RCTs directly comparing WBV and resistance training for handgrip strength. The small sample size also contributed to downgrade for imprecision, as the total number of participants did not reach the optimal information size. Regarding publication bias in the WBV versus control comparison, Egger's test indicated no evidence of publication bias in the acute model (*p* = 0.435), whereas a marginal signal was observed in the chronic model (*p* = 0.113). Nevertheless, it is important to recognise that statistical methods for detecting publication bias have limitations, and the possibility of unpublished negative findings cannot be entirely ruled out. A funnel plot could not be produced for the WBV versus resistance training comparison because of the limited number of studies.

### 4.2 Strengths and Limitations

To minimise review bias, only RCTs were included. Another strength was the comprehensive search strategy, which included nine databases, two clinical trial registries, and the reference lists of all included studies and prior systematic reviews, reducing the likelihood of missing eligible trials. However, although no language restrictions were applied and an extensive search was conducted across major biomedical databases and trial registries, the search strategy may not have captured all relevant non‐English studies or grey literature. Therefore, some potentially eligible studies may have been overlooked. A further limitation is that the main analysis did not stratify by health status, vibration intensity, or body position, although targeted subgroup analyses were conducted to address this.

Another limitation relates to incomplete reporting in some of the included studies. As indicated in Table 1, certain methodological and intervention characteristics were not reported by the original authors. Specifically, one study did not clearly report participants' age range, seven did not report the type of vibration (synchronous or side‐alternating), sixteen did not report whether adverse events occurred, two did not report the percentage of adherence, and thirteen did not report the percentage of compliance. These instances were recorded as “not reported” (NR) in the table. Such missing information may limit the interpretation of certain study characteristics and intervention parameters.

Future research should prioritise larger, well‐designed randomized controlled trials to clarify the specific conditions under which WBV may produce clinically meaningful improvements in handgrip strength. In particular, future studies should further investigate chronic protocols (approximately ≥ 10–12 weeks), higher vibration frequencies (> 20 Hz), and exercise positions involving direct hand contact with the vibration stimulus, as these were the conditions under which more pronounced within‐group effects were observed in the exploratory subgroup analyses.

## 5 Conclusions

Based on moderate‐certainty evidence, acute WBV interventions produced no significant effect on handgrip strength. Based on very low‐certainty evidence, chronic WBV interventions yielded a statistically significant but small improvement compared with inactive or sham controls, the clinical relevance of which appears limited when considered in isolation. Exploratory subgroup analyses identified conditions associated with more pronounced within‐group effects in chronic interventions, including healthcare populations, high‐frequency protocols, and direct hand contact with the vibration stimulus. No conclusion could be drawn regarding potential differences between WBV and resistance training (very low‐certainty evidence). Further well‐designed RCTs are required to clarify the specific conditions under which WBV may produce clinically meaningful improvements in handgrip strength, particularly in populations with reduced baseline grip strength.

## Author Contributions

**Julien Dines Labarrere:** data curation, formal analysis, visualization, writing – original draft. **Raphael Gonçalves de Oliveira:** conceptualization, project administration, validation, writing – review and editing. **André Luiz de Campos Pessoa:** investigation, methodology, visualization, writing – original draft. **Danúbia Cunha Sá‐Caputo:** investigation, methodology, visualization, writing – original draft. **Mario Bernardo‐Filho:** data curation, formal analysis, writing – original draft. **Liszt Palmeira de Oliveira:** methodology, project administration, supervision, validation, writing – review and editing.

## Ethics Statement

The authors have nothing to report.

## Conflicts of Interest

The authors declare no conflicts of interest.

## Supporting information


Supporting Information S1

## Data Availability

The data that support the findings of this study are available from the corresponding author upon reasonable request.

## References

[msc70246-bib-0001] Ahn, J. Y. , H. Kim , and C. B. Park . 2019. “Effects of Whole‐Body Vibration on Upper Extremity Function and Grip Strength in Patients With Subacute Stroke: A Randomised Single‐Blind Controlled Trial.” Occupational Therapy International 2019: 5820952–5820955. 10.1155/2019/5820952. 31065236 PMC6466864

[msc70246-bib-0002] AlBaiti, S. , A. Arumugam , and N. Nawayseh . 2024. “Acute Neuromuscular Responses to Whole‐Body Vibration in Healthy Individuals: A Systematic Review.” Journal of Electromyography and Kinesiology 77: 102888. 10.1016/j.jelekin.2024.102888. 38833795

[msc70246-bib-0003] Bautmans, I. , E. Van Hees , J. C. Lemper , and T. Mets . 2005. “The Feasibility of Whole Body Vibration in Institutionalised Elderly Persons and its Influence on Muscle Performance, Balance and Mobility: A Randomised Controlled Trial [ISRCTN62535013].” BMC Geriatrics 5, no. 1: 17. 10.1186/1471-2318-5-17. 16372905 PMC1368976

[msc70246-bib-0004] Bohannon, R. W. 2019. “Minimal Clinically Important Difference for Grip Strength: A Systematic Review.” Journal of Physical Therapy Science 31, no. 1: 75–78. 10.1589/jpts.31.75. 30774209 PMC6348186

[msc70246-bib-0005] Caprioli, L. , F. Campoli , S. Edriss , et al. 2024. “Effect of Whole‐Body Vibration on Sports Performance: A Literature Review.” Engineering Methodologies for Medicine and Sports: 642–662. 10.1007/978-3-031-63755-1_47.

[msc70246-bib-0006] Casimiro, J. A. , L. E. Faria do Amaral , P. C. Ferreira , M. A. Gonçalves Melo Coelho , and V. Santos Borges . 2015. “Efeitos de um Protocolo de Exercícios Sobre a Plataforma Vibratória na Força Muscular, Equilíbrio E Desempenho de Marcha em Idosas Comunitárias.” Fisioterapia Brasil 16, no. 1: 25–31. https://research.ebsco.com/linkprocessor/plink?id=66e71b16‐4cd2‐3f9e‐88d0‐498317f0cbe7.

[msc70246-bib-0007] Cheng, F. , N. Li , J. Yang , et al. 2024. “The Effect of Resistance Training on Patients With Secondary Sarcopenia: A Systematic Review and Meta‐Analysis.” Scientific Reports 14, no. 1: 28784. 10.1038/s41598-024-79958-z. 39567607 PMC11579013

[msc70246-bib-0008] Coelho‐Oliveira, A. C. , A. C. R. Lacerda , A. L. C. de Souza , et al. 2021. “Acute Whole‐Body Vibration Exercise Promotes Favorable Handgrip Neuromuscular Modifications in Rheumatoid Arthritis: A Cross‐Over Randomized Clinical.” BioMed Research International 2021, no. 1: 9774980. 10.1155/2021/9774980. 34901282 PMC8660187

[msc70246-bib-0009] Cohen, J. 1988. Statistical Power Analysis for the Behavioral Sciences. 2nd ed. Lawrence Erlbaum Associates. 10.4324/9780203771587.

[msc70246-bib-0010] Cunha, B. L. M. , J. H. Policarpo , J. R. D. Silva , et al. 2025. “Whole Body Vibration Exercise Effects on Exercise Capacity and Muscle Strength in Long Covid‐19 Patients: A Randomized Clinical Trial.” Journal of Bodywork and Movement Therapies 44: 494–500. 10.1016/j.jbmt.2025.06.013. 40954621

[msc70246-bib-0011] Dang, H. , and Z. Chen . 2019. “Effect of Task‐Oriented Training Combined With Vibration Therapy on Upper Limb Function in Patients With Hemiplegia After Stroke.” International Journal of Clinical and Experimental Medicine 12, no. 7: 8710–8717.

[msc70246-bib-0012] Delkhoush, C. T. , R. Bagheri , H. Mashhadi Hashemi , E. Fatemy , and R. Hedayati . 2020. “The Immediate Effect of Whole Body Vibration Training on the Electromyographic Activity of Contralateral Hand Muscles; a Randomized Controlled Trial.” Journal of Bodywork and Movement Therapies 24, no. 3: 293–299. 10.1016/j.jbmt.2020.02.027. 32826003

[msc70246-bib-0013] Di Giminiani, R. , L. Fabiani , G. Baldini , G. Cardelli , A. Giovannelli , and J. Tihanyi . 2014. “Hormonal and Neuromuscular Responses to Mechanical Vibration Applied to Upper Extremity Muscles.” PLoS One 9, no. 11: e111521. 10.1371/journal.pone.0111521. 25368995 PMC4219718

[msc70246-bib-0014] Dolny, D. G. , and G. F. Reyes . 2008. “Whole Body Vibration Exercise: Training and Benefits.” Current Sports Medicine Reports 7, no. 3: 152–157. 10.1097/01.CSMR.0000319708.18052.a1. 18477873

[msc70246-bib-0015] Ebing, J. , U. Gast , C. Hauptmann , D. Felsenberg , and D. L. Belavý . 2018. “Hypertrophy and Explosive‐Reactive Functioning in Sedentary Men After 10 Weeks of Whole‐Body Vibration.” Journal of Strength & Conditioning Research 32, no. 1: 27–36. 10.1519/jsc.0000000000001728. 27893474

[msc70246-bib-0016] García‐Gutiérrez, M. T. , M. R. Rhea , and P. J. Marín . 2014. “A Comparison of Different Vibration Exercise Techniques on Neuromuscular Performance.” Journal of Musculoskeletal and Neuronal Interactions 14, no. 3: 303–310. 25198225

[msc70246-bib-0017] Genest, F. , S. Lindström , S. Scherer , M. Schneider , and L. Seefried . 2021. “Feasibility of Simple Exercise Interventions for Men With Osteoporosis—A Prospective Randomized Controlled Pilot Study.” Bone Reports 15: 101099. 10.1016/j.bonr.2021.101099. 34258330 PMC8255176

[msc70246-bib-0018] Gonçalves de Oliveira, R. , H. Coutinho , M. N. M. Martins , et al. 2023. “Impacts of Whole‐Body Vibration on Muscle Strength, Power, and Endurance in Older Adults: A Systematic Review and Meta‐Analysis.” Journal of Clinical Medicine 12, no. 13: 4467. 10.3390/jcm12134467. 37445502 PMC10342949

[msc70246-bib-0019] Goudarzian, M. , M. Rahimi , N. Karimi , et al. 2017. “Mobility, Balance, and Muscle Strength Adaptations to Short‐Term Whole Body Vibration Training Plus Oral Creatine Supplementation in Elderly Women.” Asian Journal of Sports Medicine 8, no. 1. 10.5812/asjsm.36793.

[msc70246-bib-0020] Harazin, B. , and J. Grzesik . 1998. “The Transmission of Vertical Whole‐Body Vibration to the Body Segments of Standing Subjects.” Journal of Sound and Vibration 215, no. 4: 775–787. 10.1006/jsvi.1998.1675.

[msc70246-bib-0021] Higgins, J. P. T. , J. Thomas , J. Chandler , et al. 2023. Cochrane Handbook for Systematic Reviews of Interventions. Cochrane. www.training.cochrane.org/handbook.

[msc70246-bib-0022] Hua‐Rui, L. , H. Shouliang , Y. Zhengze , et al. 2025. “Optimal Dose of Resistance Training to Improve Handgrip Strength in Older Adults With Sarcopenia: A Systematic Review and Bayesian Model‐Based Network Meta‐Analysis.” Frontiers in Physiology 16: 1564988. 10.3389/fphys.2025.1564988. 40671711 PMC12263917

[msc70246-bib-0023] Jepsen, D. B. , T. Masud , A. Holsgaard‐Larsen , S. Hansen , N. R. Jørgensen , and J. Ryg . 2020. “The Combined Effect of Parathyroid Hormone (1‐34) and Whole‐Body Vibration Exercise on Physical Performance in Osteoporotic Women (Pavos Study): A Secondary Analysis From a Randomised Controlled Trial.” BMC Sports Science, Medicine and Rehabilitation 12, no. 1: 54. 10.1186/s13102-020-00204-w. PMC7487945 32944251

[msc70246-bib-0024] Jo, N. G. , S. R. Kang , M. H. Ko , et al. 2021. “Effectiveness of Whole‐Body Vibration Training to Improve Muscle Strength and Physical Performance in Older Adults: Prospective, Single‐Blinded, Randomized Controlled Trial.” Healthcare (Basel) 9, no. 6: 652. 10.3390/healthcare9060652. 34072657 PMC8226869

[msc70246-bib-0025] Kalaoglu, E. , O. F. Bucak , M. Kokce , et al. 2023. “Whole Body Vibration Activates the Tonic Vibration Reflex During Voluntary Contraction.” Journal of Physical Therapy Science 35, no. 6: 408–413. 10.1589/jpts.35.408. 37266357 PMC10231967

[msc70246-bib-0026] Keller, B. V. , M. L. Davis , W. R. Thompson , L. E. Dahners , and P. S. Weinhold . 2013. “Varying Whole Body Vibration Amplitude Differentially Affects Tendon and Ligament Structural and Material Properties.” Journal of Biomechanics 46, no. 9: 1496–1500. 10.1016/j.jbiomech.2013.03.033. 23623311 PMC4020418

[msc70246-bib-0027] Kurt, C. , and E. Pekünlü . 2015. “Acute Effect of Whole Body Vibration on Isometric Strength, Squat Jump, and Flexibility in Well‐Trained Combat Athletes.” Biology of Sport 32, no. 2: 115–122. 10.5604/20831862.1134558. 26060334 PMC4447756

[msc70246-bib-0028] Labott, B. K. , H. Bucht , M. Morat , T. Morat , and L. Donath . 2019. “Effects of Exercise Training on Handgrip Strength in Older Adults: A Meta‐Analytical Review.” Gerontology 65, no. 6: 686–698. 10.1159/000501203. 31499496

[msc70246-bib-0029] Landis, J. R. , and G. G. Koch . 1977. “The Measurement of Observer Agreement for Categorical Data.” Biometrics 33, no. 1: 159–174. 10.2307/2529310. 843571

[msc70246-bib-0030] Lee, J. S. , C. Y. Kim , and H. D. Kim . 2016. “Short‐Term Effects of Whole‐Body Vibration Combined With Task‐Related Training on Upper Extremity Function, Spasticity, and Grip Strength in Subjects With Poststroke Hemiplegia: A Pilot Randomized Controlled Trial.” American Journal of Physical Medicine & Rehabilitation 95, no. 8: 608–617. 10.1097/phm.0000000000000454. 26829094

[msc70246-bib-0031] Łochyński, D. , D. Kaczmarek , M. J. Rędowicz , J. Celichowski , and P. Krutki . 2013. “Long‐Term Effects of Whole‐Body Vibration on Motor Unit Contractile Function and Myosin Heavy Chain Composition in the Rat Medial Gastrocnemius.” Journal of Musculoskeletal and Neuronal Interactions 13, no. 4: 430–441. 24292613

[msc70246-bib-0032] Maher, C. G. , C. Sherrington , R. D. Herbert , A. M. Moseley , and M. Elkins . 2003. “Reliability of the PEDro Scale for Rating Quality of Randomized Controlled Trials.” Physical Therapy 83, no. 8: 713–721. 10.1093/ptj/83.8.713. 12882612

[msc70246-bib-0033] Martin, B. J. , and H. S. Park . 1997. “Analysis of the Tonic Vibration Reflex: Influence of Vibration Variables on Motor Unit Synchronization and Fatigue.” European Journal of Applied Physiology 75, no. 6: 504–511. 10.1007/s004210050196. 9202946

[msc70246-bib-0034] Moreira‐Marconi, E. , F. D. Carla , S. M. Danielle , et al. 2019. “Whole Body Vibration and Auriculotherapy Improve Handgrip Strength in Individuals With Knee Osteoarthritis.” Journal of Traditional Chinese Medicine 39, no. 5: 707–715. 32186121

[msc70246-bib-0035] Morel, D. S. 2017. Acute Effects of whole‐body Vibration Exercise on Handgrip and Muscular Activity of Flexor Digitorum Superficialis and Wrist Extensor Muscles in Militaries from Brazilian Army. [Master's Thesis]. Universidade do Estado do Rio de Janeiro. https://www.bdtd.uerj.br:8443/handle/1/8813.

[msc70246-bib-0036] Morel, D. S. , P. J. Marín , E. Moreira‐Marconi , C. F. Dionello , and M. Bernardo‐Filho . 2018. “Can Whole‐Body Vibration Exercises in Different Positions Change Muscular Activity of Upper Limbs? A Randomized Trial.” Dose‐Response 16, no. 4: 1559325818804361. 10.1177/1559325818804361. 30305808 PMC6176545

[msc70246-bib-0037] Page, M. J. , J. E. McKenzie , P. M. Bossuyt , et al. 2021. “The PRISMA 2020 Statement: An Updated Guideline for Reporting Systematic Reviews.” Bmj 372: n71. 10.1136/bmj.n71. 33782057 PMC8005924

[msc70246-bib-0038] Pearcey, G. E. P. , S. Alizedah , K. E. Power , and D. C. Button . 2021. “Chronic Resistance Training: Is it Time to Rethink the Time Course of Neural Contributions to Strength Gain?” European Journal of Applied Physiology 121, no. 9: 2413–2422. 10.1007/s00421-021-04730-4. 34052876

[msc70246-bib-0039] Pessoa, M. F. , D. C. Brandão , R. B. de Sá , et al. 2018. “Whole‐Body Vibration Increases Cardiopulmonary Performance in the Elderly: A Randomized Double‐Blind Clinical Trial.” Topics in Geriatric Rehabilitation 34, no. 4: 245–250. 10.1097/tgr.0000000000000201.

[msc70246-bib-0040] Pouyafar, M. , R. Askari , S. A. Hoseini Kakhk , M. Damavandi , and A. Maleki . 2021. “Comparing the Effects of Eight Weeks of Whole Body Vibration Exercise Combined With Rope Skipping at Two Different Intensities on Physical Performance of Older Men: A Randomized Single‐Blind Clinical Trial.” Iranian Journal of Ageing 16, no. 3: 376–395. 10.32598/sija.2021.16.3.2885.2.

[msc70246-bib-0041] Qiu, B. , Z. Wang , M. Yin , et al. 2025. “Effects of Whole‐Body Vibration Training on Muscle Performance in Healthy Women: A Systematic Review and Meta‐Analysis of Randomized Controlled Trials.” PLoS One 20, no. 5: e0322010. 10.1371/journal.pone.0322010. 40445930 PMC12124539

[msc70246-bib-0042] Rittweger, J. 2010. “Vibration as an Exercise Modality: How it May Work, and What its Potential Might be.” European Journal of Applied Physiology 108, no. 5: 877–904. 10.1007/s00421-009-1303-3. 20012646

[msc70246-bib-0043] Santin‐Medeiros, F. , J. P. Rey‐López , A. Santos‐Lozano , C. S. Cristi‐Montero , and N. Garatachea Vallejo . 2015. “Effects of Eight Months of Whole‐Body Vibration Training on the Muscle Mass and Functional Capacity of Elderly Women.” Journal of Strength & Conditioning Research 29, no. 7: 1863–1869. 10.1519/jsc.0000000000000830. 26102257

[msc70246-bib-0044] Santos, L. M. M. , A. C. C. Oliveira , S. F. Fonseca , et al. 2020. “Whole‐Body Vibration Exercise in Different Postures on Handgrip Strength in Healthy Women: A Cross‐Over Study.” Frontiers in Physiology 11: 469499. 10.3389/fphys.2020.469499. 33536927 PMC7848817

[msc70246-bib-0045] Schünemann, H. , J. Brożek , G. Guyatt , and A. Oxman . 2013. GRADE Handbook for Grading Quality of Evidence and Strength of Recommendations. GRADE Working Group. https://gdt.gradepro.org/app/handbook/handbook.html.

[msc70246-bib-0046] Sievänen, H. , S. Karinkanta , P. Moisio‐Vilenius , and J. Ripsaluoma . 2014. “Feasibility of Whole‐Body Vibration Training in Nursing Home Residents With Low Physical Function: A Pilot Study.” Aging Clinical and Experimental Research 26, no. 5: 511–517. 10.1007/s40520-014-0206-2. 24633589

[msc70246-bib-0047] Sire, A. , L. Lippi , A. Ammendolia , et al. 2021. “Physical Exercise With or Without Whole‐Body Vibration in Breast Cancer Patients Suffering From Aromatase Inhibitor‐Induced Musculoskeletal Symptoms: A Pilot Randomized Clinical Study.” Journal of Personalized Medicine 11, no. 12: 1369. 10.3390/jpm11121369. 34945841 PMC8707128

[msc70246-bib-0048] Souza, A. L. C. , V. A. Mendonça , A. C. Coelho de Oliveira , et al. 2020. “Whole Body Vibration in the Static Modified Push‐Up Position in Untrained Healthy Women Stimulates Neuromuscular System Potentiating Increased Handgrip Myogenic Response.” Journal of Bodywork and Movement Therapies 24, no. 4: 233–238. 10.1016/j.jbmt.2020.06.021. 33218516

[msc70246-bib-0049] Souza, H. C. M. , M. F. Pessoa , R. Dos Santos Clemente , et al. 2022. “Inspiratory Muscle Training in Addition to Whole Body Vibration for Functional and Physical Outcomes in Pre‐Frail Older Women: A Randomized Controlled Trial.” Age and Ageing 51, no. 4: afac083. 10.1093/ageing/afac083. 35397159

[msc70246-bib-0050] Soysal, P. , C. Hurst , J. Demurtas , et al. 2021. “Handgrip Strength and Health Outcomes: Umbrella Review of Systematic Reviews With Meta‐Analyses Of Observational Studies.” Journal of Sport and Health Science 10, no. 3: 290–295. 10.1016/j.jshs.2020.06.009. 32565244 PMC8167328

[msc70246-bib-0051] Spain, L. , L. Yang , J. M. Wilkinson , and E. McCloskey . 2021. “Transmission of Whole Body Vibration—Comparison of Three Vibration Platforms in Healthy Subjects.” Bone 144: 115802. 10.1016/j.bone.2020.115802. 33309990

[msc70246-bib-0052] Stania, M. , G. Juras , K. Słomka , D. Chmielewska , and P. Król . 2016. “The Application of Whole‐Body Vibration in Physiotherapy—A Narrative Review.” Physiology International 103, no. 2: 133–145. 10.1556/036.103.2016.2.1. 28639859

[msc70246-bib-0053] Su, Y. C. , and S. F. Chang . 2024. “Effects of Whole‐Body Vibration Training on Improving Physical Function, Cognitive Function, and Sleep Quality for Older People With Dynapenia in Long‐Term Care Institutions: A Randomized Controlled Study.” Applied Sciences 14, no. 15: 6830. 10.3390/app14156830.

[msc70246-bib-0054] Tan, X. , G. Jiang , L. Zhang , D. Wang , and X. Wu . 2023. “Effects of Whole‐Body Vibration Training on Lower Limb Muscle Strength and Physical Performance Among Older Adults: A Systematic Review and Meta‐Analysis.” Archives of Physical Medicine and Rehabilitation 104, no. 11: 1954–1965. 10.1016/j.apmr.2023.04.002. 37169245

[msc70246-bib-0055] Tankisheva, E. , I. Jonkers , S. Boonen , et al. 2013. “Transmission of Whole‐Body Vibration and its Effect on Muscle Activation.” Journal of Strength & Conditioning Research 27, no. 9: 2533–2541. 10.1519/JSC.0b013e31827f1225. 23222086

[msc70246-bib-0056] Tian, X. , S. Fu , J. He , R. Ma , and R. Shi . 2025. “Whole‐Body Vibration Training Improves Muscle Mass and Strength in Older Adults Through Intra‐ and Extra‐Muscular Pathways.” Frontiers in Cell and Developmental Biology 13: 1643478. 10.3389/fcell.2025.1643478. 41210259 PMC12591996

[msc70246-bib-0057] Timón, R. , A. González‐Custodio , N. Gusi , and G. Olcina . 2024. “Effects of Intermittent Hypoxia and Whole‐Body Vibration Training on Health‐Related Outcomes in Older Adults.” Aging Clinical and Experimental Research 36, no. 1: 6. 10.1007/s40520-023-02655-w. 38280022 PMC10821964

[msc70246-bib-0058] Torvinen, S. , P. Kannu , H. Sievänen , et al. 2002. “Effect of a Vibration Exposure on Muscular Performance and Body Balance. Randomized Cross‐Over Study.” Clinical Physiology and Functional Imaging 22, no. 2: 145–152. 10.1046/j.1365-2281.2002.00410.x. 12005157

[msc70246-bib-0059] Torvinen, S. , P. Kannus , H. Sievänen , et al. 2002. “Effect of Four‐Month Vertical Whole Body Vibration on Performance and Balance.” Medicine & Science in Sports & Exercise 34, no. 9: 1523–1528. 10.1097/00005768-200209000-00020. 12218749

[msc70246-bib-0060] Torvinen, S. , P. Kannus , H. Sievänen , et al. 2003. “Effect of 8‐Month Vertical Whole Body Vibration on Bone, Muscle Performance, and Body Balance: A Randomized Controlled Study.” Journal of Bone and Mineral Research 18, no. 5: 876–884. 10.1359/jbmr.2003.18.5.876. 12733727

[msc70246-bib-0061] Torvinen, S. , H. Sievänen , T. A. Järvinen , M. Pasanen , S. Kontulainen , and P. Kannus . 2002. “Effect of 4‐min Vertical Whole Body Vibration on Muscle Performance and Body Balance: A Randomized Cross‐Over Study.” International Journal of Sports Medicine 23, no. 5: 374–379. 10.1055/s-2002-33148. 12165890

[msc70246-bib-0062] Vaishya, R. , A. Misra , A. Vaish , N. Ursino , and R. D'Ambrosi . 2024. “Hand Grip Strength as a Proposed New Vital Sign of Health: A Narrative Review of Evidences.” Journal of Health, Population and Nutrition 43, no. 1: 7. 10.1186/s41043-024-00500-y. 38195493 PMC10777545

[msc70246-bib-0063] Wang, Z. , Z. Wei , X. Li , Z. Lai , and L. Wang . 2022. “Effect of Whole‐Body Vibration on Neuromuscular Activation and Explosive Power of Lower Limb: A Systematic Review and Meta‐Analysis.” PLoS One 17, no. 12: e0278637. 10.1371/journal.pone.0278637. 36473014 PMC9725163

[msc70246-bib-0064] Wong, R. M. Y. , P. Y. Wong , C. Liu , et al. 2025. “Vibration Therapy as an Intervention for Trochanteric Hip Fractures—A Randomized Double‐Blinded, Placebo‐Controlled Trial.” Journal of Orthopaedic Translation 51: 51–58. 10.1016/j.jot.2025.01.002. 39926341 PMC11802369

[msc70246-bib-0065] Zhu, Y. Q. , N. Peng , M. Zhou , et al. 2019. “Tai Chi and Whole‐Body Vibrating Therapy in Sarcopenic Men in Advanced Old Age: A Clinical Randomized Controlled Trial.” European Journal of Ageing 16, no. 3: 273–282. 10.1007/s10433-019-00498-x. 31543722 PMC6728405

[msc70246-bib-0066] Zhuang, M. , Y. Gu , Z. Wang , X. He , and N. Chen . 2025. “Effects of 12‐Week Whole‐Body Vibration Training Versus Resistance Training in Older People With Sarcopenia.” Scientific Reports 15, no. 1: 6981. 10.1038/s41598-025-91644-2. 40011687 PMC11865505

