# Supplementary material for: Effectiveness of Whole‐Body Vibration Therapy on Handgrip Strength: A Systematic Review and Meta‐Analysis of Randomized Controlled Trials
Source: Musculoskeletal Care. 2026 Jun 29;24(3):e70246. doi: 10.1002/msc.70246 (PMC13315511; doi:10.1002/msc.70246)
Supplement: Supplementary file 1 — Supporting Information S1 [file MSC-24-e70246-s001.docx]

**Supplementary Material**

**Search for each database**

Note: To increase search sensitivity, each term was searched in all fields.

**PubMed** (pubmed.gov, 1996 to September 5, 2025).

**#1:** whole body vibration

**#2:** whole body vibrations

**#3:** whole-body vibration

**#4:** whole-body vibrations

**#5:** WBV

**#6:** vibration

**#7:** vibrations

**#8:** vibratory

**#9:** vibration therapy

**#10:** vibration training

**#11:** vibration exercise

**#12:** oscillating platforms

**#13:** vibrating platform

**#14:** vibration plate

**#15:** vibration device

**#16:** mechanical vibration

**#17:** systemic vibration

**#18:** #1 OR #2 OR #3 OR #4 OR #5 OR #6 OR #7 OR #8 OR #9 OR #10 OR #11 OR #12 OR #13 OR #14 OR #15 OR #16 OR #17

**#19:** grip strength

**#20:** hand grip strength

**#21:** handgrip strength

**#22:** hand-grip strength

**#23:** handgrip

**#24:** hand grip

**#25:** hand strength [MeSH Terms]

**#26:** palmar grip

**#27:** strength dynamomet*

**#28:** grip dynamomet*

**#29:** muscle strength [MeSH Terms]

**#30:** muscle strength dynamometer [MeSH Terms]

**#31:** #19 OR #20 OR #21 OR #22 OR #23 OR #24 OR #25 OR #26 OR #27 OR #28 OR #29 OR #30

**#32:** randomized clinical trial

**#33:** randomised clinical trial

**#34:** RCT

**#35:** randomized controlled trial*

**#36:** randomised controlled trial*

**#37:** controlled clinical trial [MeSH Terms]

**#38:** controlled trial

**#39:** clinical trial [MeSH Terms]

**#40:** random*

**#41:** random allocation [MeSH Terms]

**#42:** random process

**#43**: placebo

**#44:** randomly

**#45:** trial

**#46:** group*

**#47:** allocati*

**#48:** control group

**#49:** control

**#50:** comparison

**#51:** comparative study [Publication Type]

**#52:** #32 OR #33 OR #34 OR #35 OR #36 OR #37 OR #38 OR #39 OR #40 OR #41 OR #42 OR #43 OR #44 OR #45 OR #46 OR #47 OR #48 OR #49 OR #50 OR #51

**#53:** #18 AND #31 AND #52

**Embase** (embase.com, 1947 to September 5, 2025)

**#1:** whole body vibration/exp

**#2:** whole body vibrations

**#3:** whole-body vibration

**#4:** whole-body vibrations

**#5:** WBV

**#6:** vibration

**#7:** vibrations

**#8:** vibratory

**#9:** vibration therapy/exp

**#10:** vibration training

**#11:** vibration exercise

**#12:** oscillating platforms

**#13:** vibrating platform

**#14:** vibration plate

**#15:** vibration device

**#16:** mechanical vibration/exp

**#17:** systemic vibration

**#18:** #1 OR #2 OR #3 OR #4 OR #5 OR #6 OR #7 OR #8 OR #9 OR #10 OR #11 OR #12 OR #13 OR #14 OR #15 OR #16 OR #17

**#19:** grip strength/exp

**#20:** hand grip strength

**#21:** handgrip strength/exp

**#22:** hand-grip strength

**#23:** handgrip/exp

**#24:** hand grip

**#25:** hand strength

**#26:** palmar grip

**#27:** strength dynamomet*

**#28:** grip dynamomet*

**#29:** muscle strength/exp

**#30:** muscle strength dynamometer/exp

**#31:** #19 OR #20 OR #21 OR #22 OR #23 OR #24 OR #25 OR #26 OR #27 OR #28 OR #29 OR #30

**#32:** randomized clinical trial

**#33:** randomised clinical trial

**#34:** RCT

**#35:** randomized controlled trial*

**#36:** randomised controlled trial*

**#37:** controlled clinical trial/exp

**#38:** controlled trial

**#39:** clinical trial/exp

**#40:** random*

**#41:** random allocation/exp

**#42:** random process

**#43**: placebo

**#44:** randomly

**#45:** trial

**#46:** group*

**#47:** allocati*

**#48:** control group

**#49:** control

**#50:** comparison

**#51:** comparative study/exp

**#52:** #32 OR #33 OR #34 OR #35 OR #36 OR #37 OR #38 OR #39 OR #40 OR #41 OR #42 OR #43 OR #44 OR #45 OR #46 OR #47 OR #48 OR #49 OR #50 OR #51

**#53:** #18 AND #31 AND #52

**CENTRAL** (cochranelibrary.com, 1998 to September 5, 2025)

**#1:** whole body vibration

**#2:** whole body vibrations

**#3:** whole-body vibration

**#4:** whole-body vibrations

**#5:** WBV

**#6:** vibration

**#7:** vibrations

**#8:** vibratory

**#9:** vibration therapy

**#10:** vibration training

**#11:** vibration exercise

**#12:** oscillating platforms

**#13:** vibrating platform

**#14:** vibration plate

**#15:** vibration device

**#16:** mechanical vibration

**#17:** systemic vibration

**#18:** #1 OR #2 OR #3 OR #4 OR #5 OR #6 OR #7 OR #8 OR #9 OR #10 OR #11 OR #12 OR #13 OR #14 OR #15 OR #16 OR #17

**#19:** grip strength

**#20:** hand grip strength

**#21:** handgrip strength

**#22:** hand-grip strength

**#23:** handgrip

**#24:** hand grip

**#25:** hand strength

**#26:** palmar grip

**#27:** strength dynamomet*

**#28:** grip dynamomet*

**#29:** muscle strength

**#30:** muscle strength dynamometer

**#31:** #19 OR #20 OR #21 OR #22 OR #23 OR #24 OR #25 OR #26 OR #27 OR #28 OR #29 OR #30

**#32:** #18 AND #31 in Trials

**CINAHL** (via EBSCOhost, 1937 to September 5, 2025)

**S1:** whole body vibration

**S2:** whole body vibrations

**S3:** whole-body vibration

**S4:** whole-body vibrations

**S5:** WBV

**S6:** vibration

**S7:** vibrations

**S8:** vibratory

**S9:** vibration therapy

**S10:** vibration training

**S11:** vibration exercise

**S12:** oscillating platforms

**S13:** vibrating platform

**S14:** vibration plate

**S15:** vibration device

**S16:** mechanical vibration

**S17:** systemic vibration

**S18:** S1 OR S2 OR S3 OR S4 OR S5 OR S6 OR S7 OR S8 OR S9 OR S10 OR S11 OR S12 OR S13 OR S14 OR S15 OR S16 OR S17

**S19:** grip strength

**S20:** hand grip strength

**S21:** handgrip strength

**S22:** hand-grip strength

**S23:** handgrip

**S24:** hand grip

**S25:** hand strength

**S26:** palmar grip

**S27:** strength dynamomet*

**S28:** grip dynamomet*

**S29:** muscle strength

**S30:** muscle strength dynamometer

**S31:** S19 OR S20 OR S21 OR S22 OR S23 OR S24 OR S25 OR S26 OR S27 OR S28 OR S29 OR S30

**S32:** randomized clinical trial

**S33:** randomised clinical trial

**S34:** RCT

**S35:** randomized controlled trial*

**S36:** randomised controlled trial*

**S37:** controlled clinical trial

**S38:** controlled trial

**S39:** clinical trial

**S40:** random*

**S41:** random allocation

**S42:** random process

**S43**: placebo

**S44:** randomly

**S45:** trial

**S46:** group*

**S47:** allocati*

**S48:** control group

**S49:** control

**S50:** comparison

**S51:** comparative study

**S52:** S32 OR S33 OR S34 OR S35 OR S36 OR S37 OR S38 OR S39 OR S40 OR S41 OR S42 OR S43 OR S44 OR S45 OR S46 OR S47 OR S48 OR S49 OR S50 OR S51

**S53:** S18 AND S31 AND S52

**Web of Science** (webofscience.com, 1900 to September 5, 2025)

**1:** whole body vibration

**2:** whole body vibrations

**3:** whole-body vibration

**4:** whole-body vibrations

**5:** WBV

**6:** vibration

**7:** vibrations

**8:** vibratory

**9:** vibration therapy

**10:** vibration training

**11:** vibration exercise

**12:** oscillating platforms

**13:** vibrating platform

**14:** vibration plate

**15:** vibration device

**16:** mechanical vibration

**17:** systemic vibration

**18:** 1 OR 2 OR 3 OR 4 OR 5 OR 6 OR 7 OR 8 OR 9 OR 10 OR 11 OR 12 OR 13 OR 14 OR 15 OR 16 OR 17

**19:** grip strength

**20:** hand grip strength

**21:** handgrip strength

**22:** hand-grip strength

**23:** handgrip

**24:** hand grip

**25:** hand strength

**26:** palmar grip

**27:** strength dynamomet*

**28:** grip dynamomet*

**29:** muscle strength

**30:** muscle strength dynamometer

**31:** 19 OR 20 OR 21 OR 22 OR 23 OR 24 OR 25 OR 26 OR 27 OR 28 OR 29 OR 30

**32:** randomized clinical trial

**33:** randomised clinical trial

**34:** RCT

**35:** randomized controlled trial*

**36:** randomised controlled trial*

**37:** controlled clinical trial

**38:** controlled trial

**39:** clinical trial

**40:** random*

**41:** random allocation

**42:** random process

**43**: placebo

**44:** randomly

**45:** trial

**46:** group*

**47:** allocati*

**48:** control group

**49:** control

**50:** comparison

**51:** comparative study

**52:** 32 OR 33 OR 34 OR 35 OR 36 OR 37 OR 38 OR 39 OR 40 OR 41 OR 42 OR 43 OR 44 OR 45 OR 46 OR 47 OR 48 OR 49 OR 50 OR 51

**53:** 18 AND 31 AND 52

**SPORTDiscus** (via EBSCOhost, 1985 to September 5, 2025)

**S1:** whole body vibration

**S2:** whole body vibrations

**S3:** whole-body vibration

**S4:** whole-body vibrations

**S5:** WBV

**S6:** vibration

**S7:** vibrations

**S8:** vibratory

**S9:** vibration therapy

**S10:** vibration training

**S11:** vibration exercise

**S12:** oscillating platforms

**S13:** vibrating platform

**S14:** vibration plate

**S15:** vibration device

**S16:** mechanical vibration

**S17:** systemic vibration

**S18:** S1 OR S2 OR S3 OR S4 OR S5 OR S6 OR S7 OR S8 OR S9 OR S10 OR S11 OR S12 OR S13 OR S14 OR S15 OR S16 OR S17

**S19:** grip strength

**S20:** hand grip strength

**S21:** handgrip strength

**S22:** hand-grip strength

**S23:** handgrip

**S24:** hand grip

**S25:** hand strength

**S26:** palmar grip

**S27:** strength dynamomet*

**S28:** grip dynamomet*

**S29:** muscle strength

**S30:** muscle strength dynamometer

**S31:** S19 OR S20 OR S21 OR S22 OR S23 OR S24 OR S25 OR S26 OR S27 OR S28 OR S29 OR S30

**S32:** randomized clinical trial

**S33:** randomised clinical trial

**S34:** RCT

**S35:** randomized controlled trial*

**S36:** randomised controlled trial*

**S37:** controlled clinical trial

**S38:** controlled trial

**S39:** clinical trial

**S40:** random*

**S41:** random allocation

**S42:** random process

**S43**: placebo

**S44:** randomly

**S45:** trial

**S46:** group*

**S47:** allocati*

**S48:** control group

**S49:** control

**S50:** comparison

**S51:** comparative study

**S52:** S32 OR S33 OR S34 OR S35 OR S36 OR S37 OR S38 OR S39 OR S40 OR S41 OR S42 OR S43 OR S44 OR S45 OR S46 OR S47 OR S48 OR S49 OR S50 OR S51

**S53:** S18 AND S31 AND S52

**LILACS** (lilacs.bvsalud.org, 1986 to September 5, 2025)

**Title, Abstract and Subject:** ((whole body vibration) OR (whole body vibrations) OR (whole-body vibration) OR (whole-body vibrations) OR (WBV) OR (vibration) OR (vibrations) OR (vibratory) OR (vibration therapy) OR (vibration training) OR (vibration exercise) OR (oscillating platforms) OR (vibration plate) OR (mechanical vibration) OR (systemic vibration therapy) OR (vibration device)) AND ((grip strength) OR (hand grip strength) OR (handgrip strength) OR (hand-grip strength) OR (handgrip) OR (hand grip) OR (hand strength) OR (palmar grip) OR (strength dynamomet*) OR (grip dynamomet*) OR (muscle strength)) AND ((randomized clinical trial) OR (randomised clinical trial) OR (RCT) OR (randomized controlled trial) OR (randomized controlled trials) OR (randomised controlled trial) OR (randomised controlled trials) OR (controlled clinical trial) OR (controlled trial) OR (clinical trial) OR (randomized) OR (randomised) OR (random allocation) OR (random process) OR (placebo) OR (randomly) OR (randomization) OR (random) OR (trial) OR (groups) OR (group) OR (allocation) OR (allocating) OR (control group) OR (control) OR (comparison) OR (comparative study))

**SciELO** (https://search.scielo.org/, 1998 to September 5, 2025)

**All fields:** ((whole body vibration) OR (whole body vibrations) OR (whole-body vibration) OR (whole-body vibrations) OR (WBV) OR (vibration) OR (vibrations) OR (vibratory) OR (vibration therapy) OR (vibration training) OR (vibration exercise) OR (oscillating platforms) OR (vibration plate) OR (mechanical vibration) OR (systemic vibration therapy) OR (vibration device)) AND ((grip strength) OR (hand grip strength) OR (handgrip strength) OR (hand-grip strength) OR (handgrip) OR (hand grip) OR (hand strength) OR (palmar grip) OR (strength dynamomet*) OR (grip dynamomet*) OR (muscle strength)) AND ((randomized clinical trial) OR (randomised clinical trial) OR (RCT) OR (randomized controlled trial) OR (randomized controlled trials) OR (randomised controlled trial) OR (randomised controlled trials) OR (controlled clinical trial) OR (controlled trial) OR (clinical trial) OR (randomized) OR (randomised) OR (random allocation) OR (random process) OR (placebo) OR (randomly) OR (randomization) OR (random) OR (trial) OR (groups) OR (group) OR (allocation) OR (allocating) OR (control group) OR (control) OR (comparison) OR (comparative study))

**PEDro** (pedro.org.au, 2000 to September 5, 2025)

**Abstract and Title:** whole body vibration AND handgrip

**ClinicalTrials.gov** and **WHO ICTRP** search strategy (to September 5, 2025)

Basic search: whole body vibration AND handgrip

**Supplementary Table 1**. Reports with full text not retrieved.

| **Author** | **Title** | **Place of Publication** |
| --- | --- | --- |
| Arghoun, 2025 | How adding Whole body vibration to Exercise affects Neck pain, Disability and Muscle performance in People withe Chronic Neck Pain | trialsearch.who.int (IRCT20250119064442N1) |
| Chen, 2025 | The Effects of Whole-Body Vertical Vibration Training at Different Frequencies on Musculoskeletal Strength and Lower Limb Motor Function in Elderly Patients with Osteosarcopenia | trialsearch.who.int (ChiCTR2500098066) |
| Coelho-Oliveira, 2025 | Clinical, Metabolic, Physical, Functional, Biochemical, Quality of Life, and Quality of Sleep Evaluation in Individuals With Metabolic Syndrome After Performing Systemic Vibratory Therapy on an Oscillating/Vibrating Platform | clinicaltrials.gov (NCT06907108) |
| Alanazi, 2024 | Effects of Whole-Body Vibration on Bone Mineral Density, Hand Grip Strength, and Vitamin D Levels in Middle-Aged Men: a Preliminary Clinical Trial | clinicaltrials.gov (NCT06644469) |
| Alanazi, 2024 | Whole Body Vibration in Middle Aged Men | clinicaltrials.gov (NCT06644469) |
| Boozari, 2024 | The effect of whole body vibration on patients with diabetic sarcopenia | trialsearch.who.int (IRCT20201128049511N6) |
| Casajús, 2024 | Effects of Bariatric Surgery and Whole-Body Vibration Training on Body Composition, Basal Metabolism, Physical Fitness, and Quality of Life in Morbidly Obese Patients | Annals of Nutrition Metabolism, v.80, n.2, p.9, 2024 |
| Dittmann, 2024 | Influence of an Alternating Whole-Body Vibration Training on Muscle Mass and Muscle Strength in Patients With Liver Cirrhosis and Sarcopenia: A Clinical Pilot Project | clinicaltrials.gov (NCT06337656) |
| Embaby, 2024 | Comparative Effectiveness of Whole- Body Vibration and Aerobic Training Among Egyptian Elderly With Sarcopenia | clinicaltrials.gov (NCT06293573) |
| Hua, 2024 | Study on body function changes and related mechanisms of whole body vibration training in healthy people and post-COVID-19 population | trialsearch.who.int (ChiCTR2400089314) |
| Li, 2024 | Biophysical and nutritional combination treatment for myosteatosis in patients with sarcopenia: a study protocol for single-blinded randomised controlled trial | BMJ Open, v.14, e074858, 2014 |
| Li, 2024 | A multicenter clinical RCT research on mechanism of frailty in the elderly and effect of intelligent rehabilitation technologies involving virtual reality and whole body vibration vs resistance training basing on multi-omics analysis | trialsearch.who.int (ChiCTR2400085102) |
| Manimmanakorn, 2024 | The effects of light exercise, whole-body vibration and weight vest on physiological variables in the elderly | trialsearch.who.int (TCTR20240529009) |
| Oliveira, 2024 | Effects of Whole Body Vibration in Different Devices on Bone and Muscle Mass in Postmenopausal Women | clinicaltrials.gov (NCT06222931) |
| Zhou, 2024 | Effects of resistance training combined with vibration training on the IGF-1/PI3K/AKT/FOXO3 axis in a population with sarcopenia | trialsearch.who.int (ChiCTR2400083643) |
| Backman, 2023 | SensiEx: A randomized pilot trial Sensorimotor training using whole body vibration exercise to reduce chemotherapy-induced peripheral neuropathy after treatment for breast cancer | Annals of Oncology, v.34, n.2, p.1246, 2023 |
| Casajús, 2023 | Supervised Whole Body Vibration Training for the Improvement of Body Composition, Microbiota and Physical Fitness After Bariatric Surgery | clinicaltrials.gov (NCT05695599) |
| Cheung, 2023 | RCT of Combination Effect of Vibration Treatment and HMB Supplementation on Myosteatosis and NMJ Degeneration | clinicaltrials.gov (NCT05525039) |
| Cunha, 2023 | The effects of Whole Body Vibration on long term COVID patients | clinicaltrials.gov (RBR-3qmk24m) |
| Lin, 2023 | Vibration Approach Functions in Upper Extremities for People After Stroke | clinicaltrials.gov (NCT05969249) |
| Lin, 2023 | Vibration Approach Functions in Upper Extremities for People After Stroke | clinicaltrials.gov (NCT05969249) |
| Malmir, 2023 | Long-Term Effects of Whole-Body Vibration on Sarcopenia in Geriatric Population | trialsearch.who.int (IRCT20230304057612N1) |
| Tuna, 2023 | The Relationship Between Whole Body Vibration and Muscle Strength and Thickness, Body Composition, Physical Performance, Balance, Kinesiophobia, Mood, Fatigue, Quality of Life, and Sleep in "Pre-frail" Individuals Over 65 Years of Age: Randomized Clinical Trial | clinicaltrials.gov (NCT06004999) |
| Irvani, 2022 | Comparison of the effect of whole body vibration before and after eccentric exercise on functional markers of delayed onset muscle soreness | trialsearch.who.int (IRCT20220713055455N1) |
| Moura, 2022 | Whole Body Vibration to Improve Functional Capacity, Muscle Strength and Thickness, Functionality and Quality of Life of Post-Covid-19 Patients | clinicaltrials.gov (RBR-8t983f7) |
| Lippi, 2022 | Whole-body vibration combined with physical exercise to treat aromatase inhibitor-induced musculoskeletal symptoms in breast cancer women: results of a pilot randomized controlled study | Cancer Research, v.82, n.4, p.10-15, 2022 |
| Liu, 2022 | Based on the principle of internal and external treatment and equal treatment of muscles and bones, the basic and clinical observation and study of the application of Traditional Chinese medicine intervention combined with physical factors and rehabilitation training in the treatment of sarcopenia -osteoporosis | trialsearch.who.int (ITMCTR2200005723) |
| Chun, 2021 | Can whole-body vibration treatment optimize cognitive function in older adults who have sarcopenia and are at risk of dementia: a randomized controlled trial | trialsearch.who.int (ChiCTR2100053912) |
| Lim, 2021 | Impact of Whole-body Vibration Training on Sarcopenia in Geriatric Hospitalized Patients | clinicaltrials.gov (NCT03695354) |
| Sakalli, 2021 | The Effect of Whole Body Vibration Training in Individuals With Post COVID - 19 | clinicaltrials.gov (NCT05119634) |
| Chun, 2020 | Effects on Whole Body Vibration of the Elderly with Diabetic Peripheral Neuropathy | trialsearch.who.int (ChiCTR2000040215) |
| Galt, 2020 | Therapeutic vibration in claudicants | Journal of Vascular Surgery, v.71, n.4, p.1346 |
| Kao, 2020 | Investigation of Impacts of Vibration on Muscular Strength and Functional Performance of Upper Limbs of Older Adults | clinicaltrials.gov (NCT04767932) |
| Liang, 2020 | Effectiveness of The Whole Body Vibration for Treating Sarcopenia in Hospitalized Patients Aged 80 Years and Older | clinicaltrials.gov (DRKS00022970) |
| Schröder, 2020 | Randomized controlled study in patients with gastrointestinal tumors on the effects of acupuncture therapy and vibration training against chemotherapy-induced polyneuropathy (CIPN) under oxaliplatin-containing chemotherapy | trialsearch.who.int (ChiCTR2000040215) |
| Bernardo Filho, 2019 | Effects of mechanical vibration generated on a vibratory platform in patients with Chronic Obstructive Pulmonary Disease | trialsearch.who.int (RBR‐72dqtm) |
| Çevik, 2019 | The Acute Responses of Muscles Properties to the Different Whole Body Vibration Training | clinicaltrials.gov (NCT04012762) |
| De Souza, 2019 | Efficacy of the association of inspiratory muscle training with whole body vibration on respiratory muscle strength, functionality, balance and physical performance in prefrail older women: A randomized double-blind clinical trial | European Respiratory Journal, v.54, n.63, 2019. |
| Kwoon-ho, 2019 | Elastic-band Resistance Exercise or Vibration Treatment With Hydroxymethylbutyrate (HMB) Supplement for Sarcopenic Older People | clinicaltrials.gov (NCT04028206) |
| Saldiran, 2019 | Immediate responses of the different whole body vibration training frequencies on muscles properties | Conference Abstract, p. S48 - S49 |
| Lee, 2019 | Effects of whole body vibration training using side-alternating vibration platform with tilt table in hospitalized older adults with sarcopenia: a randomized controlled pilot study | Aging Medicine and Healthcare, v.10, p.1-35, 2019 |
| Bernardo Filho, 2018 | Effects of vibrations generated in oscillating / vibratory Platform in young and Healthy individuals | trialsearch.who.int (RBR‐738wng) |
| Jepsen, 2018 | Effects of whole-body vibration exercise in combination with parathyroid hormone (1–34) on physical performance measures in osteoporotic women: a secondary analysis from a randomized controlled trial | European Geriatric Medicine Society, v.9, n.1, p.S99, 2018 |
| Moreira-Marconi, 2018 | Whole body vibration increase functionality in individuals with knee osteoartrhitis | Osteoporosis International, v.29, n.1, p.S523, 2018 |
| Seefried, 2018 | Evaluation of analysis for validation of efficiency of practical exercise modalities in elderly men at risk for osteoporosis | clinicaltrials.gov (DRKS00013262) |
| Seefried, 2018 | Whole Body Vibration in hemodialysis patients-Beneficial effects particularly in patients with impaired physical functions | Nieren- und Hochdruckkrankheiten, v.47, n.10, p.497-507, 2018 |
| Souza, 2018 | Effects of WBV Associated With IMT on Inflammatory Markers, Body Composition, Muscle Strength and Thickness | clinicaltrials.gov (NCT03689322) |
| Fortes, 2017 | Mechanical vibration used in Vibratory Platform after cardiac surgery | clinicaltrials.gov (RBR-3tgsqp) |
| Genest, 2017 | Combined efficacy of different exercise interventions in osteosarcopenic men. | ASBMR 2017 Annual Meeting, p.S398 |
| Machado, 2017 | Evaluation of the Effects of Whole Body Vibration Training on Functional Capacity, Muscle Strength, and Biochemical Profile in Elderly Women | clinicaltrials.gov (NCT03030456) |
| Morel, 2017 | Effects of whole body vibration exercise on handgrip strength and muscular activity of flexor digitorum superficialis on soldiers from the Brazilian Army | Journal of Science and Medicine in Sport, v.20, n.2, p.S160, 2017 |
| Of, 2017 | Effects of Whole Body Vibration Training in Patients With Interstitial Lung Disease | clinicaltrials.gov (NCT03560154) |
| Machado, 2016 | Whole Body Vibrations on Functional Capacity, Muscular Strength, and Biochemical Profile in Elders | clinicaltrials.gov (NCT03030456) |
| 2015 | Effects of Stochastic Whole-body Vibration Physiotherapy-WBV (Stochastic Resonance Physiotherapy-SRT) on Muscle Strength in Patients After Kidney Transplantation: a Pilot Study | clinicaltrials.gov (NCT02345577) |
| Ketenci, 2015 | Whole Body Vibration on Muscle Strength in Patients With Postpolio Syndrome | clinicaltrials.gov (NCT04387864) |
| Neves, 2015 | Effect of training in vibrating plataform in subjects with Chronic Obstructive Pulmonary Disease | trialsearch.who.int (RBR‐3kxkzn) |
| Arampatzis, 2014 | Whole-body Vibration Physiotherapy in Kidney Transplantation | clinicaltrials.gov (NCT02345577) |
| Campbell, 2013 | Intervention to prevent falls in elderly adults living in a residential home | Journal of the American Geriatrics Society, v.61, n.8, p.1426-1427, 2013 |
| Oliveira, 2013 | Mechanical vibration strongly improves neuromuscular functions and preserves bone mass in postmenopausal osteopenic women | Osteoporosis International, v.24, n.1, p.S347, 2013 |
| Pleguezuelos. 2013 | Whole Body Vibration in Chronic Obstructive Pulmonary Disease | clinicaltrials.gov (NCT01850173) |
| Sanudo, 2013 | Effectiveness of high intensity exercise training and vibration recovery on key health outcomes and weight control in overweight/obese people | trialsearch.who.int (ACTRN12613000287730) |
| Merriman, 2012 | Is whole-body vibration an effective and save treatment choice for older adults? | Journal of Geriatric Physical Therapy, v.32, n.3, p.134–145 2001. |
| Pang, 2012 | Whole Body Vibration Exercise for Elderly With Cognitive Impairments | clinicaltrials.gov (NCT01734083) |
| Pessoa, 2012 | Effects Whole Body Vibration in Ergospirometrics Parameters, Strength And Quality of Life: A Randomised Controlled Trial | clinicaltrials.gov (NCT01704976) |
| Rogan, 2012 | SR-WBV Training for Frail Elderly in the Skilling up Stage | clinicaltrials.gov (NCT01538888) |
| Sievänen, 2012 | Effect of whole body vibration training on physical performance among institutionalized older people: a 10-wk pilot blinded randomized controlled trial | IOF-ECCEO European Congress on Osteoporosis and Osteoarthritis, v2, n.23, 2012 |
| Elmantaser, 2011 | The medium and short-term effects of sinusoidal and vertical vibratory training on the musculoskeletal and endocrine system in healthy men | 50th Annual Meeting of the ESPE, p.319, 2011 |
| Lau, 2011 | he effects of whole body vibration therapy on neuromotor performance and bone metabolism in individuals with chronic stroke: a randomized controlled trial | Dissertation/ thesis, p.274, 2011 |
| Wu, 2011 | Effects of Whole Body Vibration Training on Functional Fitness in Female Elderly | Medicine and Science in Sports and Exercise, v.43, n.1, p.519, 2011 |
| Persson, 2010 | The effects of exercise and vibration therapy on bone density and physical function in females aged 55-80 with osteoporosis | trialsearch.who.int (ISRCTN1715727) |
| Bogaerts, 2008 | The effects of long term Whole Body Vibration training in older individuals. | Isokinetics & Exercise Science, v.16, n.3, p.179, 2008 |
| Lau, 2008 | The Effects of Whole Body Vibration Exercise Training on Physical Functioning and Falls in Community-dwelling Elderly With Cognitive Impairments | clinicaltrials.gov (NCT00796237) |
| Lau, 2008 | Effects of Whole Body Vibration Training on Bone Health, Physical Fitness, and Neuromotor Performance in Individuals With Subacute Stroke: a Randomized Controlled Trial | clinicaltrials.gov (NCT00796237) |
| Lau, 2008 | Effects of Whole Body Vibration in Subacute Stroke Patients | clinicaltrials.gov (NCT00796237) |
| Zhang, 2008 | The Effects of Passive and Active Vibration Therapy on Grip Strength and Myoelectric Activity | clinicaltrials.gov (NCT00779610) |
| Vella, 2005 | Research. Whole-body vibration training: shake up clients' workouts with this low-impact training method. | IDEA Fitness Journal, 2005. |
| Boonen et al., 2004 | Effects of 6-months vibration loading and resistance training on muscle strength and hip density in postmenopausal women: A comparative trial | Osteoporosis International, v.15, p.S117-18, 2004 |

**Supplementary Table 2.** Reports excluded after reading the full text.

| **Author** | **Title** | **Place of Publication** | **Reason for exclusion** |
| --- | --- | --- | --- |
| Feyzioğlu, 2025 | Effects of vibration therapy on muscle strength, shoulder range of motion, and muscle biomechanical properties in patients with breast cancer undergoing radiotherapy | American Journal of Physical Medicine & Rehabilitation, v.104, n.4, 2025 | Intervenção |
| Formica, 2025 | The role of vibraplus on fatigue in multiple sclerosis patients:  A randomized controlled trial | Journal of Clinical Medicine, v.14, n.3990 | Desfecho |
| Aboaloyoun, 2024 | Effect of whole­body vibration versus kinesio tape on strength and balance in patients with diabetic peripheral neuropathy | Fizjoterapia polska, v.24, n.4, p.282-287, 2024 | Desfecho |
| Allnutt, 2024 | The feasibility of whole-body vibration training as na approach to improve health in autistic adults | Disabilities, v.4, p.429-443, 2024 | Desenho do estudo |
| Gómez‐Bruton, 2024 | Effects of whole‐body vibration on body composition, microbiota, cardiometabolic markers, physical fitness, and quality of life after bariatric surgery: protocol for a randomized controlled trial | Trials, v.25, n.413, 2024 | Desenho do estudo |
| Li, 2024 | Vitamin d combined with whole‐body vibration training for the treatment of osteo‐sarcopenia: study protocol for a randomized controlled trial | Trials, v.25, n.638, 2024 | Desenho do estudo |
| Guedes-Aguiar, 2023 | Effects of a single session of systemic vibratory therapy on flexibility, perception of exertion and handgrip strength in chronic obstructive pulmonary disease individuals: a quasi-experimental clinical trial | Journal or Clinicaç Medicine, v.12, n.9, p.3241,2023 | Desenho do estudo |
| Beck, 2022 | The effect of low-intensity whole-body vibration with or without high-intensity resistance and impact training on risk factors for proximal femur fragility fracture in postmenopausal women with low boné mass: study protocol for the vibmor randomized controlled trial | Trials, v.23, n.15, 2022 | Desenho do estudo |
| De Araujo, 2022 | Do two whole-body vibration amplitudes improve postural balance, gait speed, muscle strength, and functional mobility in sedentary  Older women? A crossover randomized controlled trial | Journal of Bodywork & Movement Therapies, v.32, p.143-148, 2022 | Desfecho |
| Kienberger, 2022 | Effects of whole body vibration in postmenopausal osteopenic women on bone mineral density, muscle strength, postural control and quality of life: the t‐bone randomized trial | European Journal of Applied Physiology, v.122, p.2331-2342, 2022 | Desfecho |
| Lu, 2022 | Effects of vibration training vs. Conventional resistance training among community-dwelling older people with sarcopenia:  Three-arm randomized controlled trial protocol | Frontiers in Aging Neuroscience, v.14, p.905460, 2022. | Desenho do estudo |
| Taani, 2022 | Effect of semi‐recumbent vibration exercise on muscle outcomes in older adults: a pilot randomized controlled clinical trial | BMC Geriatrics, v.22, n.335, 2022 | Intervenção |
| Waheed, 2022 | Whole-body Vibration, in Addition to Balance Exercise, Shows Positive Effects for Strength and Functional Ability in Patients with Diabetic Peripheral Neuropathy: A Single-blind Randomized Controlled Trial | Journal of Diabetology, v.12, n.4, p.1-9, 2022. | Desfecho |
| Saucedo, 2021 | Effects of controlled whole-body vibration training on functional performance among healthy older adults: a 6-week pilot study | Journal of Aging Research and Lifestyle, v.10, 2021 | Desfecho |
| Yang, 2021 | Effect of whole-body vibration training on physical fitness and postural control in working-age patients on haemodialysis | Journal of Rehabilitation medicine, v.4, 2021 | Desenho do estudo |
| Oliveira, 2019 | Effects of whole-body vibration versus pilates exercise on bone mineral density in postmenopausal women: a randomized and controlled clinical trial | Journal of geriatric physical therapy, v.0, n.0, 2018 | Desfecho |
| Dionello, 2018 | Efeitos do exercício de vibração de corpo inteiro na atividade neuromuscular de membros inferiores de mulheres com lúpus eritematoso sistêmico e comprometimento de massa óssea | Theses: Universidade do Estado do Rio de Janeiro, p.1-111, 2018 | Desfecho |
| Neves, 2018 | Whole body vibration training increases physical measures and quality of life without altering inflammatory-oxidative biomarkers in patients with moderate copd | Journal of Applied Physiology, v.125, n.2, p.520-528, 2018 | Desenho do estudo |
| Aksoy, 2017 | Effect of protein intake on muscle strength and hypertrophy during whole-body vibration training | Isokinetics and Exercise Science, v.25, p.235-242, 2017 | Intervenção |
| Doyle, 2017 | The utility of whole body vibration exercise in haemodialysis patients: a pilot study | Clinical Kidney Journal, v. 10, n. 6, p. 822–829, 2017 | Desenho do estudo |
| Gloeckl, 2017 | What's the secret behind the benefits of whole-body vibration  Training in patients with copd? A randomized, controlled trial | Respiratory Medicine, v.126, p.17-24, 2017 | Desfecho |
| Munakata, 2017 | Dynamic whole-body vibration training: a unique upstream treatment from the muscle to the arterial system and central hemodynamics | Hypertension Research, v.40, p.436-438, 2017 | Desfecho |
| Seefried1, 2017 | Efficacy and safety of whole body vibration in maintenance  Hemodialysis patients - a pilot study | Journal of Musculoskeletal and Neuronal Interactions, v.17, n.4, p:268-274, 2017 | Desenho do estudo |
| Dutra, 2016 | Whole-body vibration improves neuromuscular parameters and  Functional capacity in osteopenic postmenopausal women | Menopause: The Journal of The North American Menopause Society, v.23, n.8, p.870-875, 2016 | Desenho do estudo |
| Smith, 2016 | Effects of biodensity training and power plate whole-body vibration on strength, balance, and functional independence in older adults | Joumal of Aging and Physical Activity, v.24, p.139-148, 2016 | Desfecho |
| Xu, 2016 | Eight-week vibration training of the elbow flexors by force modulation: effects on dynamic and isometric strength | Journal of Strength and Conditioning Research, v.30, n.3, p.739-746, 2016 | Desfecho |
| Braz Júnior, 2015 | Whole-body vibration improves functional capacity and quality of life in patients with severe chronic obstructive pulmonary disease (copd): a pilot study | International Journal of COPD, v.10, p.125-132, 2015 | Desfecho |
| Perchthaler, 2015 | Evaluation of a six-week whole-body vibration intervention on neuromuscular performance in older adults | Journal of Strength and Conditioning Research, v.29, n.1, p.86-95, 2015 | Desfecho |
| Salhi, 2015 | Effects of Whole Body Vibration in Patients With COPD | Journal of Chronic Obstructive Pulmonary Disease, v.12, n.5, p. 525-532, 2015 | Desfecho |
| El-Shamy, 2014 | Effect of whole-body vibration on muscle strength and balance in diplegic cerebral palsy | American Journal of Physical Medicine & Rehabilitation, v.93, n.2, 2014 | População |
| Imtiyaz, 2014 | To compare the effect of vibration therapy and massage in prevention of delayed onset muscle soreness (doms) | Journal of Clinical and Diagnostic Research, v.8, n.1, p.133-136 | Desfecho |
| Kessler, 2014 | Effect of stochastic resonance whole body vibration on functional  Performance in the frail elderly: a pilot study | Archives of Gerontology and Geriatrics, v.59, p.305-311, 2014 | Desfecho |
| Li, 2014 | Efficacy of low-magnitude high-frequency vibration on preventing fall and muscle loss in community elderly | Thesis: The Chinese University of Hong Kong, p.1-200, 2014 | Desfecho |
| Saedmocheshi, 2014 | Effect of whole body vibration on muscle performance in elderly men | Medical Journal of Tabriz University of Medical Sciences and Health Services,v.36, n.5, p.34-39, 2014 | Desfecho |
| Uszynski, 2014 | The feasibility of comparing whole body vibration intervention to the same duration and dose of exercise for people with Multiple Sclerosis | Physiotherapy Practice and Research, v.35, p.75-86, 2014 | Desfecho |
| Wang, 2024 | Whole-body vibration combined with extra-load training for enhancing the strength and speed of track and field athletes | Journal of Strength and Conditioning Researchm, v.28, n.9, p.2470-2477, 2014 | Desfecho |
| Kennis, 2013 | Effects of fitness and vibration training on muscle quality: a 1-year postintervention follow-up in older men | Archives of Physical Medicine and Rehabilitation, v.94,p.910-8, 2013 | Desfecho |
| Milanese1, 2013 | Ten-week whole-body vibration training improves body composition and muscle strength in obese women | International Journal of Medical Sciences, v.10, n.3, p.307-311, 2013 | Desfecho |
| Ayiesah, 2012 | Whole-body vibration for patients with chronic obstructive pulmonary disease e ‘thoughts for clinical practice’ | Respiratory Medicine, v.106, n.5, p.755, 2012 | Desenho do estudo |
| Eftekhari, 2012 | Resistance training and vibration improve muscle strength and functional capacity in female patients with multiple sclerosis | Asian Journal of Sports Medicine, v.3, n.4, p. 279-284, 2012 | Desfecho |
| García-López,2012 | Acute effects of whole-body vibrations on balance, maximal force and perceived exertion: vertical platform versus oscillating platform | European Journal of Sport Science, v.12, n.5, p.425-430, 2012 | Desenho do estudo |
| Karakiriou, 2012 | Effects of vibration and exercise training on bone mineral density and muscle strength in post-menopausal women | European Journal of Sport Science, v.12, n.1, 81-81, 2012 | Desfecho |
| Lachance, 2012 | The effects of whole-body vibration training on upper and lower body strength in older adults | Physical and Rehabilitation Medicine, v.24, n.1-2, p.35-50, 2012 | Desenho do estudo |
| Preatoni, 2012 | The effects of whole-body vibration in isolation or combined with strength training in female athletes | Journal of Strength and Conditioning Research, v.26, n.9, p.2495-2506, 2012 | Desfecho |
| Sanudo, 2012 | Effect of whole-body vibration exercise on balance in women with fibromyalgia syndrome: a randomized controlled trial | The Journal of Alternative and Complementary Medicine, v,18, n.2, p.158-164, 2012 | Desfecho |
| Tillaar, 2012 | The effect of 6 months of whole body vibration training on  Strength in postmenopausal women | Motricidade, v.8, n.1, p.41-50, 2012 | Desfecho |
| Eider, 2011 | Effects of 8-week intermittent whole body vibration combined with sub-maximal resistance training on strength capacities in health-related training of young females | Polish Journal of Environmental Studies, v.20, n.6, p.1453-1464 | Desfecho |
| Tomas, 2011 | The use of vibration exercise in clinical populations | ACSM’s Health & Fitness Journal, v.6, n.15, p. 25-31, 2011 | Desenho do estudo |
| Sitjà-Rabert, 2011 | Whole body vibration for older persons: an open  Randomized, multicentre, parallel, clinical trial | BMC Geriatrics, v.11, n.89, 2011 | Desfecho |
| Verschueren, 2011 | The effects of whole-body vibration training and vitamin d supplementation on muscle strength, muscle mass, and bone density in institutionalized elderly women: a 6-month randomized, controlled trial | American Society for Bone and Mineral Research, v.26, n.1, p. 42-49, 2011 | Desfecho |
| Bissonnette, 2010 | The effects of a whole-body advanced vibration exercise program on flexibility, balance, and strength in seniors | Physical & Occupational Therapy in Geriatrics, v.28, n.3, 2010 | Desfecho |
| Mikheal, 2010 | Effect of standing posture during whole body vibration training on muscle morphology and function in older adults: a randomised controlled trial | BMC Geriatrics, v.10, n.74, 2010 | Desfecho |
| Sanudo, 2010 | The effect of a 6-week exercise programme and whole body  Vibration on strength and quality of life in women with fibromyalgia: a randomised study | Clinical and Experimental Rheumatology, v.63, p.40-50, 2010. | Desfecho |
| Bogaerts, 2009 | Effects of whole body vibration training on cardiorespiratory fitness and muscle strength in older individuals (a 1-year randomised controlled trial) | Age and Ageing, v.38, p.448-454, 2009 | Desfecho |
| Bogaerts, 2007 | Impact of whole-body vibration training versus fitness training on muscle strength and muscle mass in older men: a 1-year randomized controlled trial | Journal of Gerontology: Medical Sciences, v.62, n.6, p.630-635, 2007 | Desfecho |
| Hebber, 2006 | Effekte eines niedrigfrequenten ganzkörpervibrationstrainings auf  Dem galileo 2000 | Bewegungstherapie und Gesundheitssport, v.22, p.52-57, 2006. | Desfecho |
| Luo, 2005 | The use of vibration training to enhance muscle strength and power | Sports Medicine, v.35, p.23–41, 2005 | Desenho do estudo |
| Samuelson, 1889 | Influence of vibration on endurance of maximal isometric contraction influence of vibration on endurance of maximal isometric contraction | Clinical Phisiology, v.9, p.21-25, 1989 | Intervenção |

**Supplemental Figure 1.** Sensitivity analysis comparing whole-body vibration therapy vs. control groups for handgrip strength, containing only studies with low risk of bias (PEDro score ≥ 6 points): a) acute interventions; b) chronic interventions.


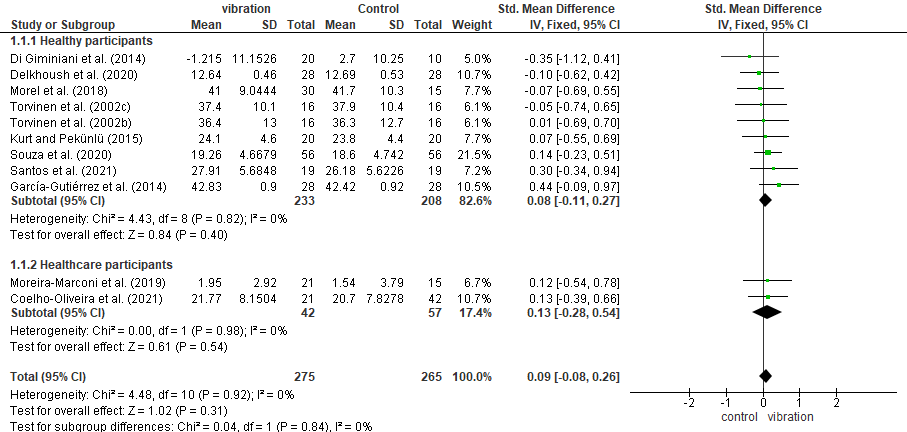


**Supplemental Figure 2.** Subgroup analysis for health status (healthy participants vs. healthcare participants) comparing whole-body vibration therapy vs. control groups for handgrip strength — acute interventions.


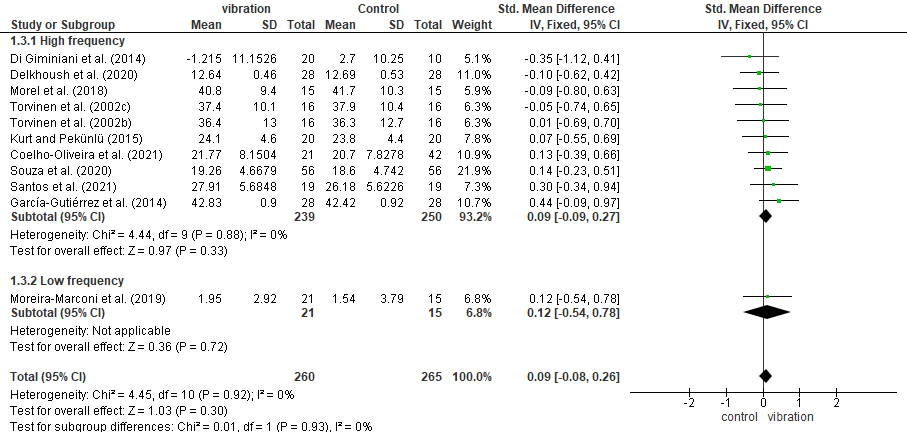


**Supplemental Figure 3.** Subgroup analysis for vibration intensity (high frequency vs. low frequency) comparing whole-body vibration therapy vs. control groups for handgrip strength — acute interventions.


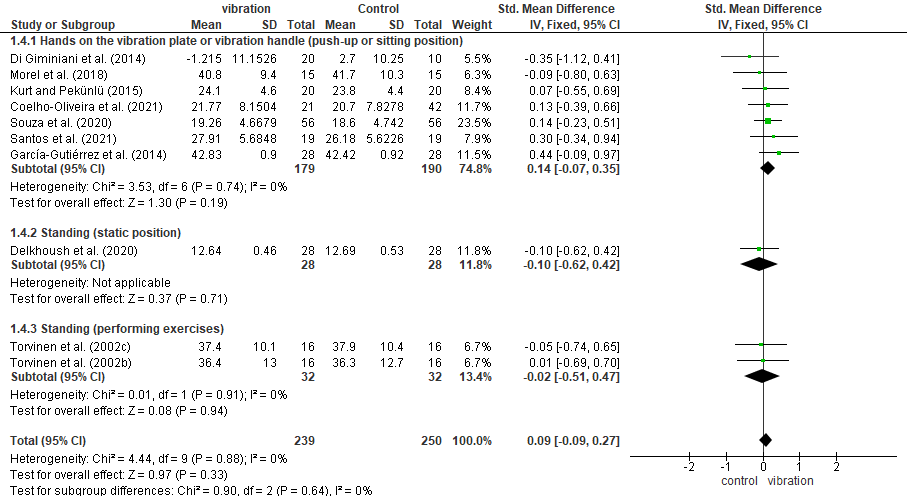


**Supplemental Figure 4.** Subgroup analysis for body positioning (static standing vs. performing standing exercises vs. with hands in direct contact with vibration) comparing whole-body vibration therapy vs. control groups for handgrip strength — acute interventions.


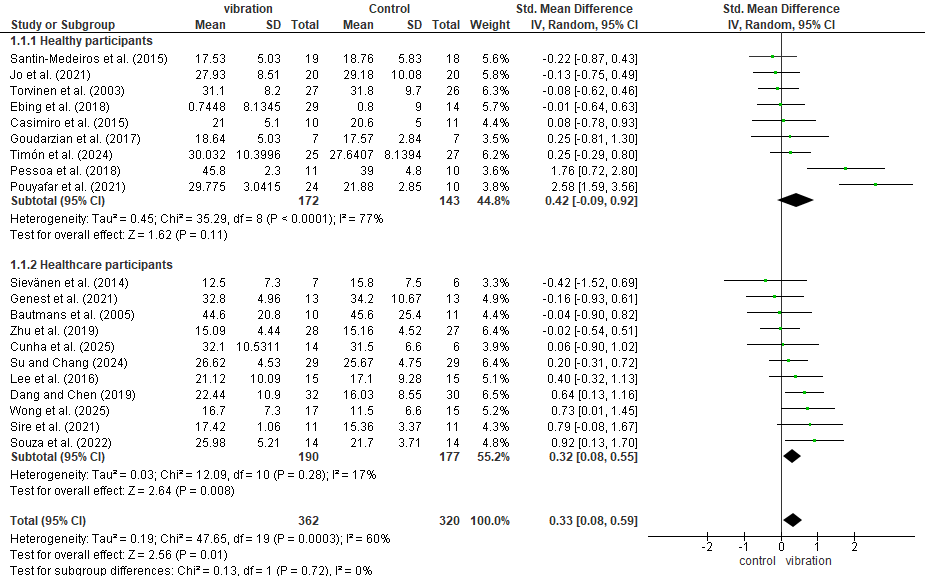


**Supplemental Figure 5.** Subgroup analysis for health status (healthy participants vs. healthcare participants) comparing whole-body vibration therapy vs. control groups for handgrip strength — chronic interventions.


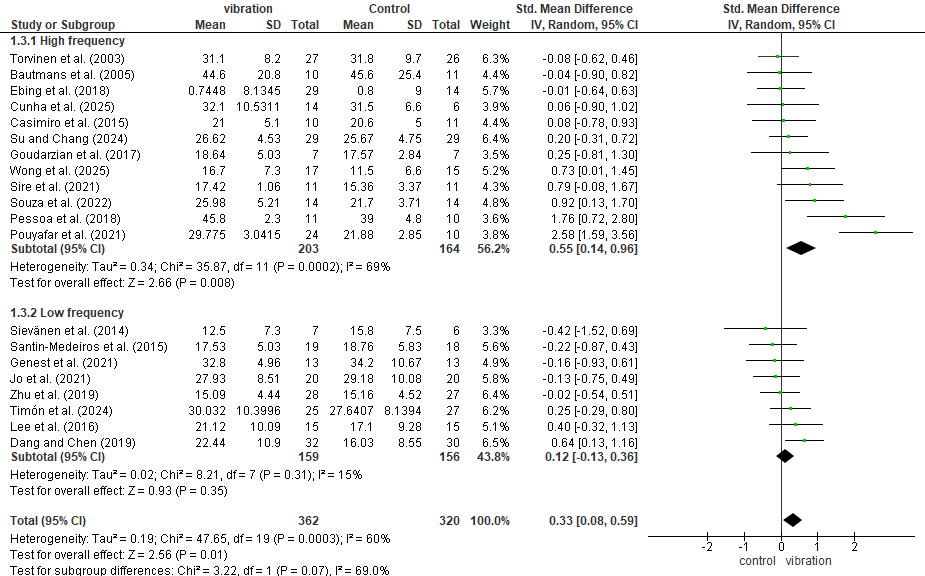


**Supplemental Figure 6.** Subgroup analysis for vibration intensity (high frequency vs. low frequency) comparing whole-body vibration therapy vs. control groups for handgrip strength — chronic interventions.


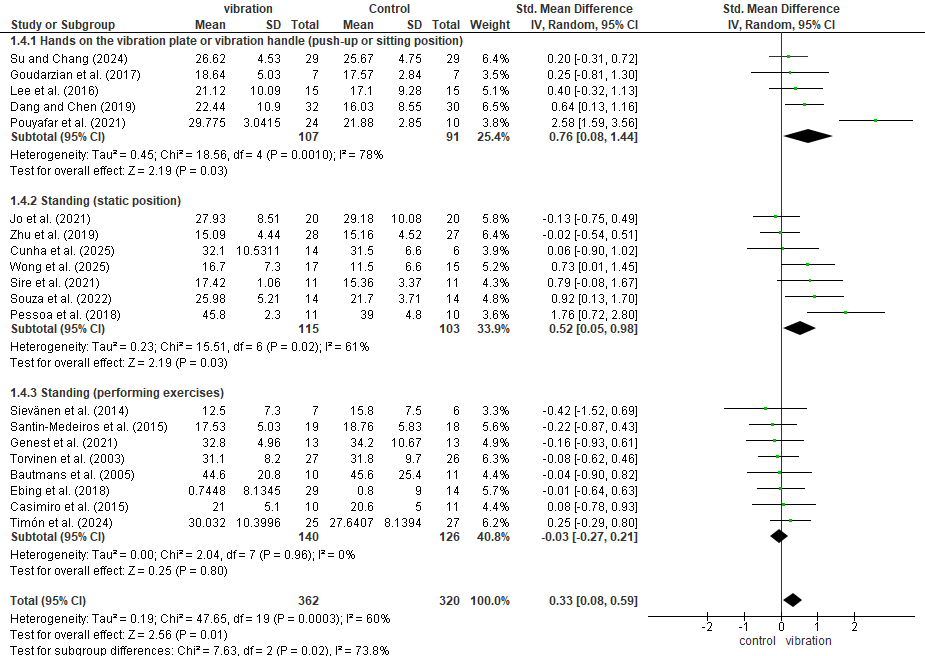


**Supplemental Figure 7.** Subgroup analysis for body positioning (static standing vs. performing standing exercises vs. with hands in direct contact with vibration) comparing whole-body vibration therapy vs. control groups for handgrip strength — chronic interventions.
